# Promoting women’s and children’s health through community groups in low-income and middle-income countries: a mixed-methods systematic review of mechanisms, enablers and barriers

**DOI:** 10.1136/bmjgh-2019-001972

**Published:** 2019-12-05

**Authors:** Lu Gram, Adam Fitchett, Asma Ashraf, Nayreen Daruwalla, David Osrin

**Affiliations:** 1 Institute for Global Health, University College London, London, UK; 2 Faculty of Life Sciences, University College London, London, UK; 3 Society for Nutrition, Education & Health Action (SNEHA), Mumbai, Maharashtra, India

**Keywords:** community mobilization, systematic review, mechanism, theory, health promotion

## Abstract

**Introduction:**

Community mobilisation through group activities has been used to improve women’s and children’s health in a range of low-income and middle-income contexts, but the mechanisms through which it works deserve greater consideration. We did a mixed-methods systematic review of mechanisms, enablers and barriers to the promotion of women’s and children’s health in community mobilisation interventions.

**Methods:**

We searched for theoretical and empirical peer-reviewed articles between January 2000 and November 2018. First, we extracted and collated proposed mechanisms, enablers and barriers into categories. Second, we extracted and synthesised evidence for them using narrative synthesis. We assessed risk of bias with adapted Downs and Black and Critical Appraisal Skills Programme checklists. We assigned confidence grades to each proposed mechanism, enabler and barrier.

**Results:**

78 articles met the inclusion criteria, of which 39 described interventions based on a participatory group education model, 19 described community-led structural interventions to promote sexual health in marginalised populations and 20 concerned other types of intervention or multiple interventions at once. We did not have high confidence in any mechanism, enabler or barrier. Two out of 15 proposed mechanisms and 10 out of 12 proposed enablers and barriers reached medium confidence. A few studies provided direct evidence relating proposed mechanisms, enablers or barriers to health behaviours or health outcomes. Only two studies presented mediation or interaction analysis for a proposed mechanism, enabler or barrier.

**Conclusion:**

We uncovered multiple proposed mechanisms, enablers and barriers to health promotion through community groups, but much work remains to provide a robust evidence base for proposed mechanisms, enablers and barriers.

**PROSPERO registration number:**

CRD42018093695.

**Key questions**

**​What is already known?**

- Community mobilisation through group activities can improve women’s and children’s health in a range of low-income and middle-income contexts.

**What are the new findings?**

- To our knowledge, this is the first systematic review of the mechanisms, enablers and barriers to health promotion through community mobilisation.
- Our study uncovered 15 proposed mechanisms and 12 proposed enablers or barriers to health promotion, but found insufficient evidence for high confidence in any particular mechanism, enabler or barrier.

**What do the new findings imply?**

- Future community mobilisation researchers need to strengthen the evidence base for not just intervention outcomes, but also intervention contexts and processes.

## Introduction

Community mobilisation interventions have been used successfully worldwide to improve maternal and newborn health,^1^ promote healthy sexual behaviours among sex workers^2^ and prevent intimate partner violence.^3^ Community mobilisation for health has long been of interest to policy-makers, funding agencies and practitioners as a means of addressing cultural, societal and environmental barriers to attaining health.^4^ It can be seen as a process based on principles of bottom–up leadership and empowerment through which local groups of individuals collaborate with external agents in identifying, prioritising and tackling the causes of ill health.^4^ This process is thought to be key to facilitating the uptake, scalability and sustainability of health programmes,^5^ improving the fit between programme objectives and local needs,^6^ and enabling citizens to hold public service providers to account.^7^

In Nepal, India, Malawi and Bangladesh, participatory women’s groups have been used to promote maternal and newborn health by employing a trained peer facilitator to lead group members through a cycle of prioritising, planning and implementing strategies to address local health problems.^1^ A meta-analysis of trials found that this approach was associated with a 20% population-level reduction in neonatal mortality.^1^ Community mobilisation programmes in India have worked with sex workers to organise collectives to promote sexual health through a combination of rights-based advocacy, peer-led support and behaviour change communication.^8^ In South Africa, community mobilisation through self-help groups has jointly addressed HIV/AIDS infection and violence against women, using a combination of economic programming and participatory education.^9^

The complex nature of community mobilisation often poses problems for evaluation within standard biomedical frameworks^10^: ‘Good’ community mobilisation is highly adapted to an ecological niche and may vary substantially from place to place. It allows participants substantial freedom to decide on their own project goals and implementation strategies, making outcomes emergent and intrinsically unpredictable. The very principles of bottom–up leadership and empowerment that are thought to make community mobilisation effective make it difficult to predict how novel contexts may react to its introduction. This raises questions about the cross-cultural generalisability of research on community mobilisation.^10^

A consensual remedy for the challenge of generalisability has often been the development of conceptually clear and empirically supported theory of how such interventions work,^11^ an approach recognised by proponents of realist evaluation,^12^ theory-driven evaluation^13^ and standard paradigms^14^ alike. That said, community mobilisation research has been criticised repeatedly for its lack of attention to theory and a perceived black box approach to evaluation in which intervention contexts and mechanisms are neither theorised nor measured.^2 10 15 16^ This lack of theory-informed evaluation has left researchers struggling to understand why interventions work in some contexts, but not in others, why health impacts are not larger or smaller than those observed, or what to expect when aspects of an intervention are changed.^10 15^ Heterogeneous interventions have been labelled as community based and participatory, even where participation was limited or tokenistic,^2^ and funding for community health initiatives has been limited by policy-makers’ uncertainty about their added value.^17^

To address this lack of empirically supported theory, we conducted a mixed-methods systematic review of mechanisms, enablers and barriers to health promotion through community mobilisation. Given the heterogeneity of interventions,^2^ we focused the review on community mobilisation interventions using groups to achieve their objectives. This large set of interventions involves groups of lay community members, women or men in regular meetings to learn about a target health issue and take action to address it. We defined mechanisms as processes that ‘intervene between the delivery of program service and the occurrence of outcomes of interest’ (p. 49), which specifically concern community members’ response to intervention delivery.^18^ We defined enablers and barriers as features of the physical and social environment—including the design of the intervention itself—that modify the ability of the intervention to produce its target outcomes.^19^

Our review sought to answer two main research questions:

1. What mechanisms, enablers and barriers have global health researchers proposed to explain the impact of community mobilisation interventions through groups on women’s and children’s health in low-income and middle-income countries?
2. What is the evidence on the roles of the proposed mechanisms, enablers and barriers in influencing women’s and children’s health in the same intervention contexts?

## Methods

### Overall review design

We developed methods a priori, described them in a PROSPERO protocol^20^—which also provides further details on review methods–and followed PRISMA (Preferred Reporting Items for Systematic Reviews and Meta-Analyses) reporting guidelines where relevant.^21^ We conducted the review in the following stages:

- We searched for and extracted studies for inclusion in the review.
- We extracted and collated proposed mechanisms, enablers and barriers to health promotion in the included studies.
- We extracted evidence relating specifically to the proposed mechanisms, enablers and barriers uncovered in the previous step from the included studies.
- We conducted quality appraisal of the extracted evidence.
- We used the results from the quality appraisal and the evidence extraction in an evidence synthesis.

### Data sources

We consulted a search librarian for databases and search terminology. We searched PubMed, Web of Science, Scopus and ProQuest for articles published in peer-reviewed journals. We chose these databases to include a broad spectrum of global health, social science and multidisciplinary outlets. PubMed and Web of Science provided articles primarily from the health and natural sciences. Scopus and ProQuest included social science and multidisciplinary outlets. We handsearched the reference lists of relevant articles and tracked citations in Google Scholar.

Table 1 shows the search terms. These were combined using and to produce a final query. We applied our search terms to all fields. We consulted past reviews of community mobilisation to develop our search terms.^2 15 22 23^ We developed them so as to cover articles which (1) described interventions aiming at community mobilisation, (2) involved community groups and (3) had a health focus. We included only articles in English and excluded studies from high-income settings according to World Bank classification. We limited the search to articles published between January 2000 and November 2018. We excluded books, posters and conference papers. We excluded grey literature as our research focused on theories of community mobilisation in academic discourse.

**Table 1:** Search terms

Search domain	Query
Community mobilisation	“social mobilisation” OR “social mobilization” OR “community mobilisation” OR “community mobilization”
Use of community groups	“village club*” OR “village group*” OR “community group*” OR “community-based group*” OR “neighbourhood group*” OR “neighborhood group*” OR “men's group*” OR “women's group*” OR “mixed group*” OR “mixed-sex group*” OR “adolescent group*” OR “youth group*” OR “youth club*” OR “care group*” OR “support group*” OR “advocacy group*” OR “citizen group*” OR “citizen's group*” OR “interest group*” OR “stakeholder group*” OR “self-help group*” OR “mother* group*” OR “father* group*” OR “health committee*” OR “health club*” OR “health group*” OR “action group*” OR “problem-solving group” OR “learning group*” OR “training group*” OR “group deliberation” OR “dialogue group*” OR “discussion group*” OR “dialogue meeting*” OR “discussion meeting*” OR “community meeting*” OR “village meeting*” OR “neighbourhood meeting*” OR “neighborhood meeting*”
Health focus	violen* OR health OR illness OR disease OR disorder OR infect* OR injury OR accident OR well-being OR biomedical* OR medical* OR medicine OR HIV

In choosing search terms, we faced challenges. The term ‘community mobilisation’ does not have a unified definition and little agreement exists on the relationship between it and its many sister constructs: community engagement, involvement, inclusion, consultation, participation, building, coalition, organisation, development, capacity, capability, resilience, power or empowerment.^2 7 15 16 22^ For example, some researchers consider the term ‘community engagement’ to denote less intensive interventions than ‘community mobilisation’,^24^ while others disagree.^25^ We did not want to attempt the contentious task of defining the difference between community mobilisation and all its sister constructs and deciding which category individual interventions belonged to. We could not simply include all group-based interventions as this would fail to exclude classroom-style health education interventions that did not aim to empower in the spirit of community mobilisation.^26^ We, therefore, chose to include only community and social mobilisation as search terms, followed by manually screening articles for mention of community groups. Previous reviews of community-related constructs have similarly included a limited number of search terms.^2 15 17 22 23 25^

### Inclusion and exclusion criteria

We included all study designs as we expected the theory for the review to come from a broad range of publications, including review articles synthesising findings from disparate studies into overarching theories, protocol articles proposing theories of change for an intervention, and formative research for new interventions. We included studies presenting theory or evidence of a mechanism, enabler or barrier to improving women’s or children’s health through community mobilisation with community groups. We defined women’s or children’s health as women’s sexual or reproductive health, maternal, newborn, child or adolescent health, or prevention of violence against women or children. We included all target populations involving women or children, including subpopulations such as female sex workers, transgender women or orphans.

We excluded articles that did not study women’s or children’s health, did not evaluate, review or conduct formative research for an intervention, did not discuss interventions involving community groups, or did not discuss any proposed mechanisms, enablers, or barriers to improving women’s or children’s health through their intervention. We also excluded articles in which community groups were not used for a health promotion purpose (eg, community-based maternal death review and audit), were not open to general members of a target population (eg, family groups, health committees, restricted organisations or federations), or were only mobilised for a single meeting (eg, ad hoc community meetings, workshops or training events).

### Search and retrieval

One reviewer (LG) conducted database searches and imported articles into Covidence,^27^ an online platform for systematic review management. LG screened abstracts and titles for articles studying any intervention involving community groups open to general members of a target population. LG read the full-text versions of articles passing abstract and title screening for fit with inclusion and exclusion criteria, including the presence of any theory, discussion, or evidence of mechanisms, enablers or barriers.

### Theory extraction

LG and a second reviewer (AA or AF) independently read each included study and extracted key information (country, health domain, target population, type of article, type of intervention, role of community groups and complementary intervention components). In the same process, LG and AA or AF extracted proposed mechanisms, barriers or enablers of health improvement, and any theories, concepts or models referenced related to a proposed mechanism, barrier or enabler. LG met with each second reviewer to compare the extracted information and resolve discrepancies, with judgement deferred to DO and ND if consensus was not reached.

LG entered a summary of the resulting consensus mechanism, barrier or enabler into a matrix. LG imported the consensus matrix into MaxQDA 2018 qualitative analysis software and collated the proposed mechanisms, barriers and enablers into overarching mechanisms, barriers and enablers. Throughout the analysis process, LG discussed his codes with AA, AF, DO and ND to ensure analytic rigour and reduce the influence of his position.

### Evidence extraction

LG rereviewed the included articles to extract qualitative or quantitative evidence concerning the collated mechanisms, enablers or barriers. LG only included primary studies and excluded all review, protocol, methods and theory articles. However, LG included one meta-regression,^1^ as it provided a type of quantitative evidence—variation in intervention impact across seven randomised controlled trials in seven different geographical contexts—that could not have been obtained through a single primary study. LG entered the resulting evidence into a matrix indicating for each code, which studies provided evidence for or against it, and whether this evidence was qualitative or quantitative.

We considered qualitative or quantitative studies to provide evidence ‘for’ a mechanism, if the study found that either the intervention produced the mechanism, or the mechanism produced an intervention outcome, or both. We considered studies to provide evidence ‘against’ a mechanism if they failed to find evidence of the above. We considered studies to provide evidence ‘for’ an enabler or barrier if they found the ability of the intervention to produce its target outcomes was affected by the enabler or barrier. We considered studies to provide evidence ‘against’ an enabler or barrier if they failed to find such evidence. A column was included for studies providing mixed evidence. A second reviewer (DO or ND) checked each entry in this matrix for 10% of the studies, including the judgement of whether the piece of evidence supported or contradicted a proposed mechanism, enabler or barrier. We found no major discrepancies.

### Risk of bias assessment

LG and AF independently conducted risk of bias assessment for all articles providing evidence concerning a mechanism, enabler or barrier. They met to establish consensus in case of disagreements and deferred judgement to DO and ND if consensus was not reached. We adapted standard methods for risk of bias assessment to the context of assessing evidence for mechanism, enablers or barriers. We used the Critical Appraisal Skills Programme checklist for qualitative studies^28^ and the Downs and Black checklist for quantitative studies.^29^ For mixed-methods studies, we assessed quantitative and qualitative components separately. In each instance, we classified studies according to the evidence presented for mechanisms, enablers or barriers rather than for intervention outcomes. For example, one randomised controlled trial presented exclusively qualitative process evaluation data as evidence for mechanisms,^30^ and we classified it as qualitative. We did not conduct an assessment of publication bias given the diversity of possible mechanisms, enablers and barriers to consider.

### Evidence synthesis

LG conducted the evidence synthesis following WHO guidelines.^31 32^ We used an integrated design,^33^ in which qualitative and quantitative study results were analysed together. The aim of this analysis was to allow the findings to confirm, extend or refute each other. We did not transform qualitative into quantitative data or vice versa.^33^ We took an epistemological position that both types of findings were able to speak to one another without having to be transformed. We used a narrative synthesis^34^ to assimilate study findings into separate summary conclusions for each hypothesised mechanism, enabler or barrier due to the heterogeneity of the evidence base.

In line with recent Cochrane reviews on mechanisms and contextual modifiers,^35 36^ we assigned each mechanism, enabler or barrier a confidence grade using our prior evidence extraction and risk assessment. We adapted the CERQual approach.^35 36^ For each mechanism, enabler or barrier, we considered: (1) the methodological limitations of the studies that fed into the finding, (2) the extent to which studies painted a coherent picture across contexts and (3) the extent to which studies showed clear links with a health outcome or health behaviour. We scored mechanisms, enablers and barriers supported by studies of high quality and high coherence with a tight connection to health as having ‘high confidence’. If all three dimensions scored low, we assigned a ‘low confidence’. In all other cases, we assigned a ‘medium confidence’ score.

## Results

### Data retrieval

**Figure 1:**
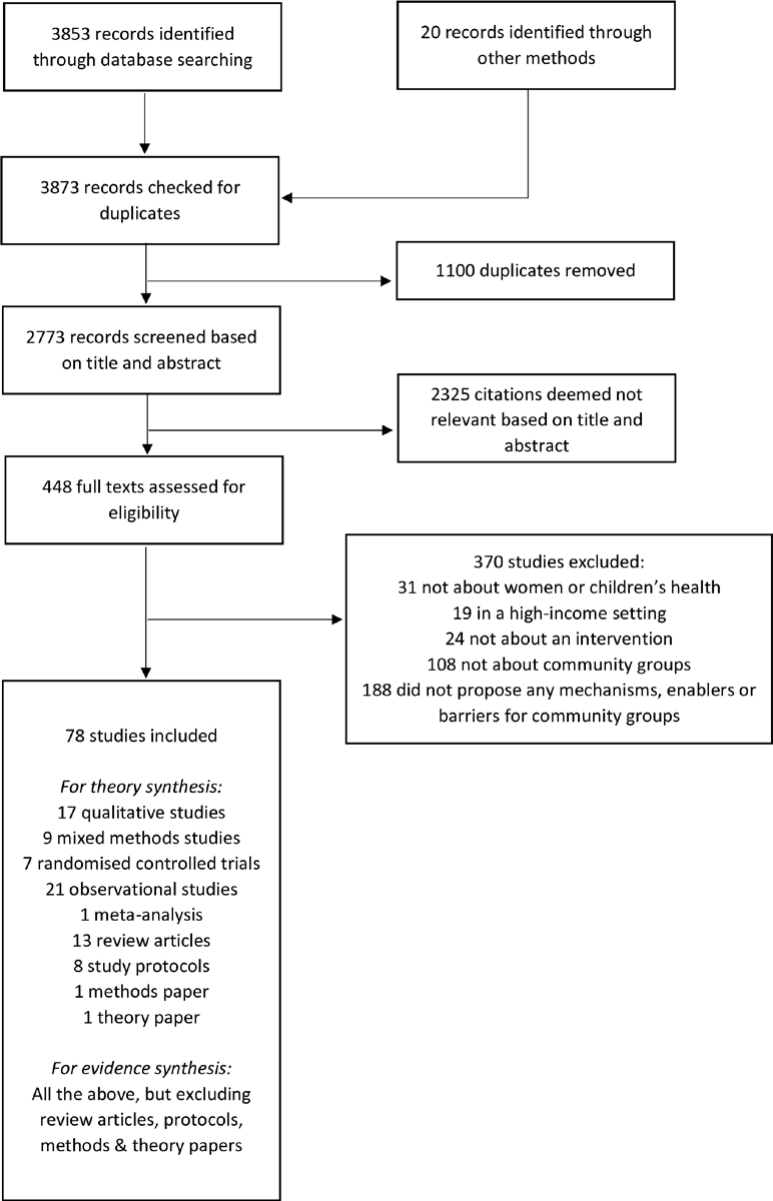
PRISMA flowchart for study extraction.

Merging database searches across PubMed, Web of Science, Scopus and ProQuest yielded 3853 records. Handsearch and citation tracking yielded 20 more (figure 1). After removing duplicates, we screened abstracts and titles of 2773 records, of which 2325 were deemed not relevant. This left 448 articles for full text assessment. We excluded 370 studies. 19 studies took place in a high-income setting, 31 did not concern women’s or children’s health and 24 did not concern an intervention. 108 did not describe interventions involving community groups, while 188 did not propose any mechanisms, enablers, or barriers for intervention effect through community groups. We included 78 studies for data extraction.

Of these, 17 were qualitative (22%), 9 used mixed-methods (12%), 7 were randomised controlled trials (9%), 21 were observational (27%), 13 were review articles (17%), 1 was a meta-analysis (1%), 8 were study protocols (10%), 1 was a methodological paper (1%) and 1 was a theory paper (1%). As described in the Methods section, we excluded review articles, study protocols, methods papers and theory papers from evidence extraction and synthesis, but included them in theory extraction.

### Characteristics of included papers

Online supplementary table 1 summarises characteristics of individual papers. 36 were concerned with maternal or child health (46%), 29 with women’s sexual health (37%)—18 of which focused on HIV prevention among commercial sex workers (62%)—and 23 with violence against women and girls (29%), of which 14 focused on intimate partner violence (61%), 3 on female genital cutting (13%) and 2 on violence against sex workers (9%). One article studied child maltreatment (1%). In terms of study locations, 55 were in South Asia (70%), 34 sub-Saharan Africa (44%), 6 Latin America and the Caribbean (8%), 5 East Asia (6%) and 1 North Africa and the Middle East (1%). Forty-two studies were in India (54%), 17 in Nepal (22%), 13 in Bangladesh (17%) and 13 in South Africa (17%).

10.1136/bmjgh-2019-001972.supp1 Supplementary data

### Types of intervention

Nineteen studies (24%) described community-led structural interventions, primarily associated with sex worker rights programmes in the Avahan initiative in India.^8^ These involved a combination of peer-led outreach, provision of sexual health services and community mobilisation. The community mobilisation component involved providing safe spaces for sex workers to gather and identify issues to tackle as a collective, forming and building capacity of community-based groups and developing groups into larger self-sustaining organisations. Thirty-nine (50%) studies described participatory group education interventions, primarily associated with interventions to promote maternal and child health^1^ or prevent intimate partner violence.^37^ These involved engaging groups of local residents in dialogue, reflection and action based on Freirean principles.^38^ The aim was to further awareness of the social roots of ill health and spur action towards social change. Of these studies, 22 described pure participatory group education interventions, while 18 combined participatory group education with complementary interventions such as microfinance, resource transfers or livelihoods training, home visits, mass media, quality improvement at health facilities or provision of health services. Ten studies (13%) described neither a community-led structural intervention nor a participatory group education intervention, but instead described financial self-help groups, social accountability initiatives or care groups. Ten studies (13%) reviewed combinations of interventions.

### Risk of bias

Assessments of individual studies are provided in online supplementary tables 2 and 3 for qualitative and quantitative evidence, respectively. Of 28 studies involving qualitative evidence, only 18 clearly aimed to explore mechanisms, enablers or barriers. Eighteen studies provided insufficient detail on their sampling strategy; frequently, we could not assess whether the study had compared a sufficiently broad range of participants to draw conclusions about mechanisms, enablers or barriers. Eighteen studies did not adequately consider the relationship between researchers and participants; often, researchers did not consider the effect of power differences between interviewers and respondents on interview dynamics and respondent answers. Even among the studies that did consider this, all of them simply commented that respondents might have been motivated to provide pleasing answers to the interviewer, without further analysis. However, most studies used appropriate data collection and analysis methods, took ethical issues into account and had a clear statement of study findings (19+ studies).

10.1136/bmjgh-2019-001972.supp2 Supplementary data

10.1136/bmjgh-2019-001972.supp3 Supplementary data

Of 36 studies involving quantitative evidence, 23 clearly aimed to explore mechanisms, enablers or barriers. Twenty-two studies had applied psychometric tests to validate multi-item measures where appropriate, such as calculating Cronbach’s alpha. No studies tried to account for social desirability bias in their measurement of social constructs, for example, by using list randomisation or randomised response surveys.^39^ Only nine studies took adequate account of confounding with 12 taking partial account by adjusting for basic demographic and socioeconomic factors, but not psychosocial confounders such as household agency. Only seven studies took appropriate account of missing data; many did not conduct a missing data analysis. Only two studies presented any kind of mediation or interaction analysis for a proposed mechanism, enabler or barrier. One did a path analysis of intervention effect on health outcomes through mediator variables.^40^ One did a meta-regression of results from randomised controlled trials.^1^ However, most studies adequately described their sample, used representative sampling procedures, provided CIs or SEs, used appropriate statistical tests and had a clear statement of results (19+ studies).

### Mechanisms

Table 2 lists proposed mechanisms extracted from publications, along with descriptions of each mechanism based on what individual articles proposed and discussed. Table 3 lists studies providing evidence for and against the proposed mechanisms. The complete extraction matrix for evidence is in online supplementary table 4. We broadly divided the set of possible mechanisms into mobilisation activities and mediating capacities. Mobilisation activities were conducted by group facilitators, group members and community members. Mediating capacities were developed in group and community members during the intervention. In accordance with their nested nature—individuals living in households within communities—we organised these capacities by socioecological level.^41^ Interestingly, none of the categories of proposed mechanism were specific to a particular country, intervention or health outcome.

10.1136/bmjgh-2019-001972.supp4 Supplementary data

**Table 2:** Proposed mechanisms

Mobilisation activities	Description	Discussed by
Group participation	Community members attend group meetings and become members of their community group.	1 30 42–47 49 55 61 62 67 69 70 76 80 107–113
Group deliberation	Group members engage in open, critical dialogue with each other and their facilitator, identify shared problems, decide on and set goals, develop collective solutions and evaluate past initiatives.	2 4 22 25 37 42 43 48 49 51–55 57 60 67 75–77 79–81 87 107 109 112–119
Individual acts of information sharing	Sharing information within the group and across social networks in the wider community.	15 30 37 44 46 48 54 57–62 71 86 107
Informal social support	Mutual emotional, economic and practical support; referral for health problems; crisis support and protection from violence and harassment.	2 4 15 22 30 37 40 44–48 51 55 58 63–66 72 73 77 81 82 84 85 111 114 119–122
Collective action	Group and community members carry out collective action to address shared health issues, such as protest, self-help or resource mobilisation.	1 4 8 15 23 25 30 37 42 44 46–48 52 56 58 60 61 65–67 69–74 78–81 85 109 111 113 116 121–124
**Mediating capacities**	**Description**	**Discussed by**
*Individual level*		
Critical consciousness	Capacity to critically examine one’s own and others' beliefs and values, relate one’s own vulnerability to wider social forces and question the immutability of everyday reality.	4 37 42 43 46–49 55 60 72 79 107 116 118 124 125
Attitudes and norms relevant to a health issue	Concern for a health problem; perceived value of addressing a health problem; perceived social disapproval of harmful behaviour; critical personal attitude to harmful behaviour.	8 49 51 53 55 61 62 64 71 75 77–79 110 113 119 121 123
Self-concept	A sense of agency, purpose and inspiration in one's own life; a sense of confidence and self-efficacy; self-worth and self-esteem; a sense of entitlement to basic rights; improved self-knowledge.	4 8 25 40 46–48 55 60 63 65 66 70 72 75 79–82 84 85 107 109 110 113 114 116 118 122 123
Technical knowledge/skills	Knowledge of the epidemiology of a health problem, knowledge of effective ways to address it, knowledge of legal rights and entitlements.	4 22 48 52 53 60 62 71 73 75 77 78 107 108 112 113 122 125
Practical knowledge/skills	Leadership, negotiation and communication skills; problem formulation, decision-making and problem-solving skills; ability to translate theory into action.	4 25 37 40 49 61–63 70 79–81 84 115 116
*Household level*		
Women’s position in the household	Status, respect, support and decision-making power in the household for women.	1 59 72 76 79 81 82 109 110 113 119 120 122 123
*Collective level*		
Social cohesion	A shared sense of belonging, identity and trust; well connected, mutually supportive social networks; cohesion among group or community members.	2 8 15 23 25 40 44–47 50 52 59 61 68 71 74 77 79 80 83 84 86 109–111 113–115 119 121 123 124
Civic attitudes and norms	Shared attitudes and norms around informal social support and collective action; shared belief in the collective efficacy of one’s group or community.	2 8 15 25 40 44–46 50 52 64–66 68 71 73 77 80 84 85 108 111 121 124
Self-governance	Sense of ownership over process of addressing a health issue; presence of initiative and leadership; effective management of own resources; ability to discuss, agree and make decisions as a group.	8 15 23 25 46 51 61 63 64 68 71 73 78 83 86 111 113 122 126 127
Institutional linkage	Dialogue and partnership between community and institutions; better accountability and responsiveness of institutions to the community; links between community groups and institutions	4 8 25 47 48 51 56 61 63 64 67 68 74 78 115 120 126

**Table 3:** Evidence concerning mechanisms

	Evidence for		Evidence against		Mixed evidence		Overall
**Mobilisation activities**	Qualitative	Quantitative	Qualitative	Quantitative	Qualitative	Quantitative	**Confidence**
Group participation	42 43 46	1 44 45	None	None	None	47	Low
Group deliberation	37 48–50 52–56	None	None	None	None	None	Medium
Informal information sharing	37 44 46 48 54 57–61	57 58 62	None	None	30	None	Low
Informal social support	37 44 46 48 52 57	47 58 65 66	None	64	30	40 45	Low
Collective action	46 48 56 60 61 67–69 71	46 47 56 65 66 72 85	None	None	30 37 50 58 70	None	Low
**Mediating capacities**	Qualitative	Quantitative	Qualitative	Quantitative	Qualitative	Quantitative	**Overall confidence**
*Individual level*							
Critical consciousness	37 48 50 51 53	None	None	None	49	None	Low
Attitudes and norms relevant to a health issue	59 71	62 71 75	None	None	44	76	Low
Self-concept	37 58 60 72	47 75	None	40	44	66	Low
Technical knowledge/skills	48 53 56 60 61	56 62 71 77 78	None	None	None	None	Medium
Practical knowledge/skills	None	None	None	None	None	None	None
*Household level*							
Women’s position in the household	59 72	72	None	81 82	54	76	Low
*Collective level*							
Social cohesion	46 52 60	45–47	43 59 83	86	50 55	40	Low
Civic attitudes and norms	None	44 46 65 85	None	None	None	40 45 64 66 84	Low
Self-governance	68 78	None	83	None	46	73 86	Low
Institutional linkage	48 56 61 67	56	None	None	None	74	Low

For most of the mechanisms, the current state of evidence gave us low confidence in their role in community mobilisation. We had medium confidence in two mechanisms (group deliberation and technical knowledge and skills). We had no confidence in one mechanism (practical knowledge and skills) as we could not find a study providing evidence for it. No mechanism was purely studied for a specific country, intervention or health outcome.

### Mobilisation activities

*Group participation*: Evidently, attendance is required for groups to form at all.^42^ Lower attendance means that fewer community members will be exposed to behaviour change communication^43^ and provided opportunities to develop peer support^44^ and collective capacity.^45^ Inconsistent attendance may also complicate the development of trust and social cohesion,^43 46^ as each group meeting has a new mix of participants with their own social dynamics. Two quantitative studies found group members had better health outcomes than non-members^44^ and contexts with high levels of attendance showed greater health improvement than contexts with low levels,^1^ but another study found mixed evidence for an association between health and group participation at both individual and ecological levels.^47^

*Group deliberation*: Qualitative evidence indicated that group members frequently shared in meetings experiences, concerns and opinions about both their own health issues and wider problems in the community,^37 48–55^ a process that might facilitate the development of critical consciousness.^37^ It might also help group members pool individual pieces of knowledge into a more accurate, collective understanding of health^53^ and decide on and plan solution strategies.^48 56^

*Informal information sharing*: Multiple qualitative studies found that group members shared information and advice with other members and their wider social network.^30 37 44 46 48 54 57–61^ In quantitative studies, over 90% of community members reported receiving health information from a group member,^57^ and over 90% of group members shared health information with others.^58 62^ One study reported qualitative findings that this might not necessarily happen in contexts of low baseline social cohesion, such as urban informal settlements.^30^ No study provided evidence that informal information sharing actually contributed to changes in health outcomes or behaviour.

*Informal social support*: Many qualitative studies found group members engaged in informal acts of financial, emotional or practical support for community members. These included helping others access health services,^30 48 56 57^ counselling and negotiating with family members,^37 46 48 57^ and standing up to violence.^44^ In quantitative studies, 46%–89% of the target population agreed that they were helped by a peer, group member or member of a community-based organisation when they had problems.^58 63^ However, quantitative evidence that the level of social support actually increased—rather than shifted from friends and relatives to group members—was mixed,^46 47 64^ as was evidence that the support improved actual health outcomes.^40 45 65 66^

*Collective action*: Multiple qualitative and quantitative studies found group and community members engaging in organised collective action such as awareness-raising and education campaigns, self-organised services, collective financial schemes, meetings with leadership structures, and civic marches and protests.^30 37 46–48 56 60 61 67–71^ However, quantitative and qualitative evidence that such action was both widespread and beneficial to health was mixed. Some studies found that women did not have time to participate or that they only participated in forms of collective action that were relatively ineffectual, such as small microsavings groups that had insufficient funds to make a difference to health outcomes.^30 37 46 50 58 65 70 72^ Others found collective action was sometimes associated with improved health.^66 73 74^

### Mediating capacities

#### Individual level

*Critical consciousness*: Multiple qualitative studies described the development of critical consciousness—the ability to reflect critically on everyday reality and uncover the social roots of ill health—as a key contributor to social and behavioural change.^37 48 50^ Group meetings might have catalysed the development of critical consciousness by helping community members to understand the widespread, shared nature of their personal problems.^51 53^ However, one study suggested that the development of critical consciousness should not be taken for granted as group facilitators might act in counterproductive ways.^49^ Instead of engaging with group members as equal partners in their own right, they sought to position themselves as ‘experts’ tasked with ensuring that members provided the ‘correct’ answers to their questions.^49^

*Attitudes and norms relevant to a health issue*: Qualitative and quantitative studies of interventions to prevent violence against women generally found reductions in accepting attitudes towards violent practices,^59 62 75 76^ but this did not hold for all violence-related attitudes.^44 76^ Two quantitative studies measured changes in subjective norms and found reductions in beliefs that partners or community members approved of violent practices.^62 75^ One study of an intervention to improve maternal health found qualitative and quantitative evidence for greater social pressure on husbands to support wives during pregnancy.^71^ We did not find a study linking changes in attitudes and norms directly with changes in health behaviours or outcomes.

*Self-concept*: Multiple qualitative studies reported increased self-confidence among group members as a result of gaining new knowledge, voicing opinions in public and connecting with people outside their household.^37 58 60 72^ Quantitative studies also found evidence for increased self-efficacy.^47 72 75^ However, the role of self-efficacy might vary by behaviour, as two studies of sex worker collectives found evidence for improved self-efficacy in dealing with clients and partners, but not with the police.^44 66^ Only one study attempted to link self-efficacy with a health behaviour or outcome.^40^ Disappointingly, it found no quantitative evidence that self-efficacy in condom use was linked with actual condom use.

*Technical knowledge and skills*: Qualitative studies found that groups enabled members to get a more accurate understanding of health by providing opportunities for them to pool individual knowledge through story-telling, visual games and other interactive learning activities.^48 53 60 61^ In turn, group members might share information with the wider community either informally or through organised collective action. One study of participatory women’s groups found that 96% of groups organised health education sessions for the community.^56^ Quantitative studies also consistently reported increases in knowledge of relevant health issues.^62 71 77 78^ However, we found no study evaluating whether increased knowledge was linked with improved health behaviour or outcomes.

*Practical knowledge and skills*: Thirteen articles discussed improvements in practical knowledge and skills as potentially part of the intervention mechanism. Examples included ‘life skills’,^79^ leadership,^25 37 40^ decision-making^80^ or skill in formulating and solving problems in general.^4 37 79 81^ However, we found no articles providing direct evidence for this.

#### Household level

*Women’s position in the household*: Two qualitative studies of an intervention to prevent intimate partner violence found evidence for greater female autonomy and respect for wives from husbands.^59 72^ However, quantitative evaluation of the same intervention showed a non-significant increase,^72^ while two quantitative studies of participatory women’s groups found little evidence for impact on household agency.^81 82^ A qualitative study of an intervention to engage fathers in group discussion found that it actually entrenched patriarchal norms by encouraging husbands to police their wives’ behaviour in accordance with advice from facilitators.^54^ We found no study linking women’s position in the household to health outcomes or behaviours in an intervention context.

#### Collective level

*Social cohesion*: Multiple studies reported qualitative evidence that group members felt that their groups were based on principles of trust, solidarity and respect,^46 52^ extended their social networks,^60^ and provided them with a new social identity.^46 50^ One quantitative study found non-significant evidence for greater community solidarity in times of crisis,^46^ another found members of sex work collectives reported a greater sense of unity with other sex workers.^47^ However, multiple qualitative studies reported limited social cohesion in community groups,^43 59^ sometimes due to pre-existing divisions^50 83^ and even loss of social cohesion^55^ when the intervention required group participants to act in non-conforming ways. Studies relating social cohesion to health outcomes reported mixed results. One found no quantitative evidence for an impact on condom use,^45^ but another path analysis suggested that social cohesion mediated impacts on condom use.^40^

*Civic attitudes and norms*: Multiple quantitative studies of sex worker programmes sought to relate ‘collective efficacy’—the belief that sex workers could work together to deal with shared problems—with intervention exposure and outcomes. The results were mixed and depended on the measure of collective efficacy,^84^ choice of outcome,^44 65 84 85^ programme exposure,^64^ confounders,^40 45^ geographical region^64^ or period.^66^ Only one study of a non-sex worker programme addressed this mechanism and found non-significant increases in belief in community support towards common goals.^46^ Surprisingly, no qualitative studies reported on this intervention mechanism.

*Self-governance*: Qualitative studies emphasised the importance of community ownership of the problem-solving process and found that extensive capacity building could create a sense of ownership.^68 78^ Capacity for self-governance may be important for community groups, as corruption, mismanagement, and leadership challenges have all been found to undermine group solidarity and mobilisation activity.^46^ However, only three studies, two quantitative and one qualitative, systematically evaluated the impact of community mobilisation on capacity for self-governance. All found that community groups were not ready for independence and had limited potential for long-term sustainability.^73 83 86^ We did not find a study linking this mechanism with health outcomes or behaviours.

*Institutional linkages*: Studies of participatory women’s groups found qualitative evidence of group members engaging with the health system through a range of means: supporting local village health committees, holding meetings with health providers, training traditional birth attendants and lobbying local government.^48 56 67^ One study found that such activities were widespread, as 96% of groups invited health workers to hold health education sessions, 71% trained traditional birth attendants and 48% lobbied government for health workers to staff mobile clinics.^56^ Another found that group members often continued to broker links between community members and the health system after the end of the intervention.^61^ However, a quantitative a study of a social accountability initiative found mixed evidence for increased feelings of trust, shared power or mutual responsibility between community members and health workers, despite the intervention being designed to improve relationships between the community and health workers.^74^ We did not find a study relating this mechanism to a health outcome or a health behaviour.

### Enablers and barriers

Table 4 shows a collation of proposed enablers and barriers. Table 5 lists studies providing evidence for and against them. The complete extraction matrix for evidence is in online supplementary file 4. We can broadly divide enablers and barriers into those that pertain to the community or to the intervention context, and further divide intervention context into intervention design and management and intervention implementation. None of the proposed enablers or barriers were specific to a particular country, intervention or health outcome.

**Table 4:** Proposed enablers and barriers

Community context	Description	Discussed by
Pre-existing poverty	Material poverty, poor access to employment and education, financial dependence on husbands or employers, insecure tenure of housing.	30 37 43 45 49 50 54 55 58 67 76 83 84 110 112 127
Supportive institutional-political context	Political will to tackle health issue, health system minimally functioning and able to respond to community concerns, lack of violent conflict, insecurity and instability.	25 59–61 71 73 74 76 78 80 111 118 127
Pre-existing social cohesion	Existing sense of belonging, identity and trust, existing social networks and community groups, history of living and working together.	22 30 50 64 70 73 83 84 121 127
Supportive pre-existing health beliefs, attitudes and norms	Existing awareness and concern with health issue, prior confidence that issue can be addressed, culture of open discussion around issue.	37 50 54 59 61 69 83 111 127
Pre-existing power hierarchies in the community	Lack of voice and decision-making power for women in the community, stigmas of sex and reproduction, power relations between men.	22 42 50 53 54 58 59 63 64 68 69 81 83 118 127
Pre-existing power hierarchies within households	General lack of female household agency; husbands forbidding wives to attend group meetings; unequal power relations between daughters-in-law and mothers-in-law.	42 43 49 50 58 59 62 67 69 79 81 82 110 114 119
**Intervention context**	**Description**	**Discussed by**
*Intervention design and management*		
Staff management	Effective recruitment, training and supervision of group facilitators; staff confidence, motivation and retention.	37 42 49 52 58 67 78 80 83 107 112 125
Incentives for participation	Cash or food transfers at group meetings; reimbursements for taxi fare; microfinance initiatives; help accessing entitlements.	37 43 48 55 58 59 70 86 87 107 108
Managing community relations	Engaging stakeholders; avoiding backlash; building relationships with community members.	2 50 52 61 78 83 87 122
*Intervention implementation*		
Respect for local people, knowledge and practices	Avoiding trying to ‘teach’ group members and being open to learning from group members; negotiating flexibly, not demanding change.	37 42 48–50 61 62 69 114 122 127
Relevant education tools	Locally accessible education materials; relevant language used; presence of a meeting agenda.	42 48–50 69 107 109
Inclusion of less powerful subpopulations	Participation of less powerful community members and equal opportunity for all to contribute to group activities	37 48 50–52 69 83 107 109 114 122

**Table 5:** Evidence concerning enablers and barriers

	Evidence for		Evidence against		Mixed evidence		Overall
**Community context**	Qualitative	Quantitative	Qualitative	Quantitative	Qualitative	Quantitative	**confidence**
Barrier: pre-existing poverty	30 43 50 55 83	None	69	None	58	Low
Enabler: supportive institutional-political context	37 61	None	None	None	None	None	Medium
Enabler: pre-existing social cohesion	50 83	None	None	None	None	None	Medium
Enabler: supportive pre-existing health beliefs, attitudes and norms	50 53 54 58 59 68	53	48 72	None	None	None	Low
Barrier: pre-existing power hierarchies in the community	50 54 69 83	None	None	None	None	None	Medium
Barrier: pre-existing power hierarchies within households	43 50 58 69	62	None	None	None	None	Medium
**Intervention context**							
*Intervention design and management*							
Enabler: staff management	42 49 58 83	None	None	None	None	None	Medium
Enabler: incentives for participation	37 43 58 59	81 86	None	None	None	None	Medium
Enabler: management of community relations	50 52 87	None	None	None	None	None	Medium
*Intervention implementation*							
Enabler: respect for local people, knowledge and practices	37 49 50	None	None	None	None	None	Medium
Enabler: relevant education tools	42 48 50 69 71	None	None	None	None	None	Medium
Enabler: inclusion of less powerful subpopulations	69	69	None	None	None	None	Medium

We had medium confidence in most of the proposed enablers and barriers, and low confidence for two barriers (pre-existing poverty and pre-existing supportive health beliefs, attitudes and norms). Apart from pre-existing social cohesion and inclusion of vulnerable subpopulations, no other enabler or barrier was purely studied for one specific country, intervention or health outcome. Evidence concerning pre-existing social cohesion only existed in India and evidence concerning inclusion of vulnerable subpopulations as an enabler only existed for participatory women’s groups in maternal and child health. We found no study directly linking enablers or barriers to intervention impact on health behaviour or outcomes.

### Community context

*Pre-existing poverty*: Qualitative studies reported high levels of pre-existing poverty in the target population impeded intervention efforts by reducing participant time for group activities,^30 43 55 83^ reducing participant social status in the community,^50 58^ disrupting solidarity through competition over resources,^43 50 83^ displacing focus on long-term social change with immediate material concerns,^30 43^ or reducing participant agency through economic dependency on others.^43^ However, poverty might also increase motivation to engage with the intervention if people perceived themselves to have greater need of it. Quantitative results from two different interventions showed that poorer and less educated women were actually more likely to attend group meetings, while qualitative results showed that better-off women perceived less need to attend.^58 69^

*Supportive institutional-political context*: One qualitative study^61^ found that local support from political groups and health staff enabled more active groups, and discussions were livelier and more productive when local health and government personnel attended meetings. Conversely, another qualitative study found that community leaders sometimes prohibited collective action that did not fall in line with their views, leading to group members giving up on the planned action.^37^

*Pre-existing social cohesion*: Two qualitative studies reported that less cohesive communities were more difficult to mobilise due to mistrust, competitiveness and social isolation.^50 83^

*Supportive pre-existing health beliefs, attitudes* *and norms*: Studies of interventions to prevent violence against women reported that pre-existing beliefs and attitudes to marriage and masculinity affected men’s motivation to participate in group meetings, engage in bystander intervention and allow their wives to participate.^54 58 59^ Stigma associated with participation in group meetings was also found to reduce group attendance.^68 69 83^ Nonetheless, studies also reported qualitative evidence that initial community resistance to tackling sensitive health issues could be overcome through continued dialogue with community members.^48 72^

*Pre-existing power relations between community members*: Multiple qualitative studies described how hierarchical power relations between community members due to inequality along lines of gender, class, age or employment obstructed intervention efforts because more powerful community members excluded less powerful ones from group meetings,^69^ challenged the authority of the less powerful to speak out in public,^50 58^ or prevented the less powerful from being reached by programme staff.^83^

*Pre-existing power hierarchies between household members*: Multiple studies found qualitative evidence of male partners and in-laws actively forbidding women from joining group meetings due to fears about women breaching seclusion norms, spreading gossip about their household, learning bad habits from others or becoming too independent.^43 50 69^ A quantitative study found spousal prohibition to be one of the most common reasons for non-participation.^62^

### Intervention context

#### Intervention design and management

*Staff management*: Multiple studies reported qualitative evidence of poor staff management affecting intervention implementation. In two studies, poor staff morale caused either individual groups or whole programmes to be abandoned.^42 58^ Another two studies found that pressure on staff to demonstrate performance on quantitative indicators and material incentives for such performance undermined sustainability, community ownership and participatory pedagogy.^49 83^

*Incentives for participation*: Qualitative and quantitative studies of interventions providing material incentives found that they dramatically increased meeting attendance,^81^ promoted programme acceptability^59^ and motivated members to join.^86^ Conversely, qualitative studies reported that lack of ability to pay for transport or forego time spent on income-earning work prevented participation in group meetings^43^ and collective action.^37 58^

*Managing community relations*: Qualitative studies reported that conscious effort to manage relationships with community members was key to trust and credibility. This involved hiring local people to convene group meetings,^48^ engaging in dialogue with community stakeholders,^52^ and providing tangible support to community members outside group meetings; for example, by helping them access entitlements.^50 87^

#### Intervention implementation

*Respect for local people, knowledge* *and practices*: Qualitative studies have repeatedly described poor relationships between staff and community members—staff lacking an ethos of open communication and participation—obstructing intervention efforts.^37 49 50^ This usually manifested as staff seeing the function of groups as sharing knowledge with women who were ‘blank’ to ensure they were able to give ‘correct’ answers and ‘achieve’ behaviour change, sometimes even through punitive measures. Conversely, negotiating peacefully and flexibly with communities rather than demanding change was found to convey respect for local views and facilitate acceptance of intervention messages.^50^

*Relevant education tools*: Multiple studies noted how the use of simple, locally appropriate, fun discussion tools such as picture cards, stories or interactive games stimulate critical thinking and enhance learning in groups.^48 50 69 71^ Conversely, where facilitators lacked a clear agenda, relevant health education tools or knowledge of appropriate language for group meetings,^42 50^ group members protested that meetings were a waste of their time and sometimes even dissolved their groups.

*Inclusion of less powerful* *sub* *populations*: A mixed-methods study concluded that socioeconomic differentials in attendance at participatory women’s group meetings were small because of active measures to include less powerful subpopulations: facilitators went door-to-door to invite and persuade poor women to attend and deliberately convened meetings close to their homes at convenient times.^69^

## Discussion

To our knowledge, this is the first mixed-methods systematic review of mechanisms, enablers and barriers to health promotion through community groups. Our review uncovered a large number of possible mechanisms, enablers and barriers, ranging from group participation to institutional linkage, from community power relations to staff and resource management. However, the number of articles proposing a mechanism, enabler or barrier exceeded the number providing evidence for it. Eleven articles proposed inclusion of marginalised populations as an enabler of intervention impact, but only one provided evidence for it. Fifteen studies proposed improved practical skills in making decisions, solving problems and assuming leadership as a mechanism, but no articles provided evidence. Thirteen studies proposed supportive institutional-political context as an enabler, but only two studies provided evidence. Our Risk of Bias assessment did not produce high confidence in any one mechanism, enabler or barrier. We are reluctant to add to this theory-evidence gap by proposing a conceptual framework of mechanisms, enablers or barriers. Nonetheless, we believe our results gesture towards issues for policy-makers and researchers to consider.

First, our review shows the complexity of successfully delivering community mobilisation interventions. Implementers might need to ensure the cooperation of local stakeholders, while simultaneously challenging unequal power structures. They might need to show respect for local values while promoting attitude and norm change. They might have to nurture community ownership over health, while promoting help-seeking from external providers. This potential need to accommodate multiple, at times conflicting, desiderata resonates with descriptions in past literature of implementers having to allow a certain degree of ‘necessary contradiction’ between ideology and practice in order for their intervention to succeed.^88^ Second, our results show that positive social processes to address health problems cannot be taken for granted. Past studies have cautioned that calls for greater community participation in health often assume that involving people in programme decisions will cause them to be empowered, without an evidential basis for this claim.^10 89 90^ Our review results support this strongly, as almost all our proposed categories of mechanism involved studies reporting evidence both for and against. Finally, our study shows how issues of power are intricately entangled in the production of health. The majority of enablers and barriers could be cast in terms of problematic distributions of power between different stakeholders, whether household members, community members or implementing agencies. The majority of mechanisms addressed the individual and collective powers of the target population to mobilise and take action. This echoes a recent review, which found power relations to be key to influencing health outcomes in community participation initiatives.^91^

Our review revealed clear gaps in the current evidence base. Fully 91% of studies were based in South Asia or sub-Saharan Africa, presenting a need for more studies in Latin America, East Asia and the Middle East. We found limited theory and evidence concerning social processes at the household level, as all the studies recovered focused on issues of gender equality and women’s empowerment. Future studies might be able to uncover more clues about household-level processes in community mobilisation if they complement a traditional conflict perspective^92^ with theoretical frameworks on topics such as family communication,^93^ family systems dynamics^94^ or family development^95^ adapted to local context. Similar to a recent systematic review of the impact of combination HIV programmes on empowerment and agency,^96^ we found studies linking mechanisms, enablers and barriers to health behaviour or outcomes rare. Although evidence may exist outside the context of community mobilisation interventions, it is important to generate evidence from specific interventions, because the intervention context itself may affect mechanisms. For example, a recent study of an intervention to prevent intimate partner violence in Uganda found that social capital was associated with bystander intervention in intervention areas, but not control areas.^97^ The researchers hypothesised that intervention areas might have established social norms disapproving of violence that allowed social capital to be translated into action against violence.

Our review suggests improvements to strengthen theory and research methods. Despite growing momentum for realist enquiry,^12^ theoretical writing tended to use linear conceptualisations of intervention mechanism akin to pure logic models.^98^ For example, a theory of change for the prevention of intimate partner violence might posit that group discussions about gender norms lead to increased awareness, which in turn causes behavioural change, which in turn improves health.^76^ This affected our review, as we sought only to evaluate mechanisms that had already been proposed by global health researchers. The many mechanisms we found mixed evidence for point to a clear need for more nuanced approaches, such as realist theory, in which mechanisms only activate when ‘firing conditions’ are met.^99^ For example, a recent theory of change of community mobilisation to prevent domestic violence in India posited that community members being willing to participate in the programme was a necessary precondition for groups with collective agency to develop.^100^ Current best practice guidelines for theories of change actually recommend listing necessary conditions for mechanisms.^101^ By moving beyond pure logic models as the theoretical basis for interventions, future researchers might develop evaluative frameworks that better capture complexity in community mobilisation.

At the level of empirical testing, our Risk of Bias assessment showed the need for more comprehensive approaches to providing empirical evidence for context and process. Few studies attempted to account for socially desirable responses from study participants despite the often explicit intention of community mobilisers to activate positive social processes of community ownership and increased social cohesion.^102 103^ Qualitative studies using a realist or critical realist ontology might benefit from reduced reliance on self-report through greater use of ethnographic observation.^104^ Quantitative studies might benefit from triangulation with techniques to correct for social desirability bias such as list randomisation and randomised response surveys.^39^ Causal evidence for a true mediating role of many proposed mechanisms was also weak. As mentioned before, evaluations rarely accounted convincingly for confounding, provided evidence of mediator impact on health outcomes or behaviour, or performed statistical mediation analysis. Future mechanism evaluations might benefit from use of quasi-experimental methods. For example, a study of political protest exploited natural random variation in turn-out at Tea Party events due to rainfall in the USA to estimate the impact of protest size on political response.^105^

### Limitations

Given our use of explicit search terms for ‘community mobilisation’ and ‘social mobilisation’, we may have missed articles that did not label their intervention as such. Some researchers describe interventions consistent with our definition of ‘community mobilisation’ under the headings ‘community participation’ or ‘community engagement’.^4 25^ As we explained, we chose our terms as a pragmatic compromise due to the need to exclude interventions that were not community mobilisation interventions in the face of disagreement in the academic literature over the precise difference—if any—between participation, engagement and mobilisation.

A sizeable proportion of studies (20%) concerned the Avahan initiative to prevent HIV/STI infections among sex workers in India. This may have weighted theory and evidence towards its assumptions and conclusions. However, all the mechanisms, enablers and barriers except two were proposed and empirically investigated for multiple countries, interventions and health outcomes. None were exclusively proposed or empirically investigated by studies from Avahan, and Avahan studies did not appear disproportionately supportive or opposed to particular mechanisms, enablers or barriers compared with studies of other interventions.

Finally, only one reviewer was involved in article screening due to resource limitations, which might have resulted in relevant articles being missed.^106^

## Conclusion

In response to past scepticism concerning the state of theory in community mobilisation research,^2 10 15 16^ we reviewed the global health literature for mechanisms, enablers and barriers to health promotion in community mobilisation interventions. Our review uncovered numerous potential mechanisms, enablers and barriers to explore. We hope that researchers and practitioners consider it a basis for developing hypotheses to investigate in their own community mobilisation interventions. In doing so, we collectively move closer towards an evidence-based theory of community mobilisation.
