# Supplementary material for: Promoting women’s and children’s health through community groups in low-income and middle-income countries: a mixed-methods systematic review of mechanisms, enablers and barriers
Source: BMJ Glob Health. 2019 Dec 5;4(6):e001972. doi: 10.1136/bmjgh-2019-001972 (PMC6936553; doi:10.1136/bmjgh-2019-001972)
Supplement: Supplementary data [file bmjgh-2019-001972supp001.pdf]

Description of individual studies

Author(s) and title	Year	Country focus	Health focus	Target population	Type of article	Type of intervention	Role of community groups	Complementary intervention components
Biradavolu et al. Structural stigma, sex work and HIV: contradictions and lessons learnt from a community-led structural intervention in southern India	2012	India	HIV/AIDS	Commercial sex workers	Qualitative study	Community-led structural intervention	*Avahan initiative:*<br>Community building & formation of sex worker community-based organisations was integral to the community mobilisation part of the intervention	Condom distribution & STI treatment<br>Advocacy with police & media<br>Social Change Agents recruited to act as peer educators & community organizers
Blankenship et al. Power, community mobilization, and condom use practices among female sex workers in Andhra Pradesh, India	2008	India	HIV/AIDS	Commercial sex workers	Cross-sectional survey	Community-led structural intervention	*Avahan initiative:*<br>Social Change Agents organize community-based organizations composed of & led by female sex workers	Condom distribution & STI treatment<br>Taking female sex workers to clinics<br>Advocacy on behalf of sex workers<br>Social Change Agents recruited to act as peer educators & community organizers
Gaikwad et al. How effective is community mobilisation in HIV prevention among highly diverse sex workers in urban settings? The Aastha intervention experience in Mumbai and Thane districts, India	2012	India	HIV/AIDS	Commercial sex workers	Cross-sectional survey	Community-led structural intervention	*Avahan initiative:*<br>Initiation of self-help groups, community-based organisations & federations of CBOs<br>Capacity building of community-based organizations on programme & financial management	Peer educators conducted health promotion activities<br>Negotiation with policy-makers on AIDS control at the state level<br>Sensitisation of police & local goons<br>Legal literacy training<br>Crisis response<br>Networking meetings between bar owners and self-help group leaders
Galavotti et al. Navigating the swampy lowland: a framework for evaluating the effect of community mobilisation in female sex workers in Avahan, the India AIDS Initiative.	2012	India	HIV/AIDS	Commercial sex workers	Theory paper	Community-led structural intervention	*Avahan initiative:*<br>Providing safe spaces for sex workers to gather<br>Support for sex workers to identify issues to tackle as a collective<br>Formalisation & ownership of community groups & networks<br>Capacity building of community-based organizations	Peer-led outreach, referral & education<br>STI & TB treatment & testing<br>Distribution of condoms & needles<br>Crisis response

Author(s) and title	Year	Country focus	Health focus	Target population	Type of article	Type of intervention	Role of community groups	Complementary intervention components
Guha et al. Risk reduction and perceived collective efficacy and community support among female sex workers in Tamil Nadu and Maharashtra, India: the importance of context	2012	India	HIV/AIDS	Commercial sex workers	Cross-sectional survey	Community-led structural intervention	*Avahan initiative:*<br>Regular training & meetings for sex workers, formation of self-help groups and community-based organisations	Peer-led outreach & skills training for sex workers
Kongelf et al. Is Scale-Up of Community Mobilisation among Sex Workers Really Possible in Complex Urban Environments? The Case of Mumbai, India	2015	India	HIV/AIDS	Commercial sex workers	Qualitative study	Community-led structural intervention	*Avahan initiative:*<br>Peer educator-led community group meetings in safe spaces<br>Training, capacity building & network strengthening of community-based groups facilitating the formation of community-based organisations	Peer-led outreach with emphasis on condom promotion & distribution and enhanced access & uptake of STI screening & treatment<br>Community crisis response
Kuhlmann et al. Investing in communities: Evaluating the added value of community mobilization on HIV prevention outcomes among FSWs in India	2014	India	HIV/AIDS	Commercial sex workers	Cross-sectional survey	Community-led structural intervention	*Avahan initiative:*<br>Drop-in centres providing safe meeting spaces for sex workers<br>Sex worker participation in formal & informal community-based organisations	Peer-led outreach & condom distribution<br>Crisis response<br>Services for STIs<br>Encouraging sex worker participation in programme activities: community mapping & outreach, membership of project committees, management of crisis response teams
Nagarajan et al. Female sex worker's participation in the community mobilization process: Two distinct forms of participations and associated contextual factors	2014	India	HIV/AIDS	Commercial sex workers	Cross-sectional survey	Community-led structural intervention	*Avahan initiative:*<br>Sex worker participation in self-help groups and formal & informal community-based organisations	Peer-led outreach, referral & education<br>Crisis response<br>Services for STIs

Author(s) and title	Year	Country focus	Health focus	Target population	Type of article	Type of intervention	Role of community groups	Complementary intervention components
Narayanan et al. Monitoring community mobilisation and organisational capacity among high-risk groups in a large-scale HIV prevention programme in India: selected findings using a Community Ownership and Preparedness Index	2012	India	HIV/AIDS	Commercial sex workers	Cross-sectional survey	Community-led structural intervention	*Avahan initiative:*<br>Capacity building of community-based groups focusing on organisational capacity and sustainability	Peer-led behaviour change communication, condom & needle distribution<br>STI treatment and referral to HIV care
Saggurti et al. Community collectivization and its association with consistent condom use and STI treatment-seeking behaviors among female sex workers and high-risk men who have sex with men/transgenders in Andhra Pradesh, India	2013	India	HIV/AIDS	Commercial sex workers	Cross-sectional survey	Community-led structural intervention	*Avahan initiative:*<br>Providing safe spaces for sex workers to gather<br>Support for sex workers to identify issues to tackle as a collective<br>Formalisation & ownership of community groups & networks<br>Capacity building of community-based organizations	Peer-led outreach, referral & education<br>STI & TB treatment & testing<br>Distribution of condoms & needles<br>Crisis response
Thomas et al. Design of a Community Ownership and Preparedness Index: using data to inform the capacity development of community-based groups	2012	India	HIV/AIDS	Commercial sex workers	Methodological paper	Community-led structural intervention	*Avahan initiative:*<br>Support for the formation of community-based groups & organisations<br>Capacity building of community-based groups to manage targeted interventions & conduct advocacy	Peer-led behaviour change communication, condom & needle distribution<br>STI treatment and referral to HIV care

Author(s) and title	Year	Country focus	Health focus	Target population	Type of article	Type of intervention	Role of community groups	Complementary intervention components
Vejella et al. Community Collectivization and Consistent Condom Use Among Female Sex Workers in Southern India: Evidence from Two Rounds of Behavioral Tracking Surveys	2016	India	HIV/AIDS	Commercial sex workers	Cohort study	Community-led structural intervention	*Avahan initiative:*<br>Providing safe spaces for sex workers to gather<br>Support for sex workers to identify issues to tackle as a collective<br>Formalisation & ownership of community groups & networks<br>Capacity building of community-based organizations	Peer-led outreach, referral & education<br>STI & TB treatment & testing<br>Distribution of condoms & needles<br>Crisis response
Bhattacharjee et al. Understanding the role of peer group membership in reducing HIV-related risk and vulnerability among female sex workers in Karnataka, India	2013	India	HIV/AIDS & violence against sex workers	Commercial sex workers	Mixed methods study	Community-led structural intervention	*Avahan initiative:*<br>Providing safe spaces for sex workers to gather<br>Support for sex workers to identify issues to tackle as a collective<br>Formalisation & ownership of community groups & networks<br>Capacity building of community-based organizations	Peer-led outreach, referral & education<br>STI & TB treatment & testing<br>Distribution of condoms & needles<br>Crisis response
Beattie et al. Reducing violence and increasing condom use in the intimate partnerships of female sex workers: study protocol for Samvedana Plus, a cluster randomised controlled trial in Karnataka state, south India	2016	India	HIV/AIDS & violence against sex workers	Commercial sex workers	Study protocol	Community-led structural intervention	Female facilitators engage female sex workers in group-based participatory reflection sessions<br>Capacity building for community-based organization & support for CBO to build alliances	Female facilitators counsel sex workers & link them to services & commodities<br>Leadership training for two female sex workers from each reflection group who demonstrate emergent leadership skills<br>One-to-one sessions with intimate partners of female sex workers<br>Couples counselling & couples events<br>Crisis management system

Author(s) and title	Year	Country focus	Health focus	Target population	Type of article	Type of intervention	Role of community groups	Complementary intervention components
Beattie et al. Community Mobilization and Empowerment of Female Sex Workers in Karnataka State, South India: Associations With HIV and Sexually Transmitted Infection Risk	2014	India	HIV/AIDS	Commercial sex workers	Cross-sectional survey	Community-led structural intervention	*Avahan initiative:*<br>Providing safe spaces for sex workers to gather<br>Support for sex workers to identify issues to tackle as a collective<br>Formalisation & ownership of community groups & networks<br>Capacity building of community-based organizations	Peer-led outreach, referral & education<br>STI treatment & testing<br>Advocacy on behalf of sex workers
Chakravarthy et al. Community mobilisation programme for female sex workers in coastal Andhra Pradesh, India: processes and their effects	2012	India	HIV/AIDS	Commercial sex workers	Cross-sectional survey	Community-led structural intervention	*Avahan initiative:*<br>Providing safe spaces for sex workers to gather<br>Support for sex workers to identify issues to tackle as a collective<br>Formalisation & ownership of community groups & networks<br>Capacity building of community-based organizations	Peer-led outreach, referral & education<br>STI treatment & testing
Parimi et al. Mobilising community collectivisation among female sex workers to promote STI service utilisation from the government healthcare system in Andhra Pradesh, India	2012	India	HIV/AIDS	Commercial sex workers	Cross-sectional survey	Community-led structural intervention	*Avahan initiative:*<br>Providing safe spaces for sex workers to gather<br>Support for sex workers to identify issues to tackle as a collective<br>Formalisation & ownership of community groups & networks<br>Capacity building of community-based organizations	Peer-led outreach, referral & education<br>STI treatment & testing

Author(s) and title	Year	Country focus	Health focus	Target population	Type of article	Type of intervention	Role of community groups	Complementary intervention components
Shaikh et al. Empowering communities and strengthening systems to improve transgender health: outcomes from the Pehchan programme in India	2016	India	HIV/AIDS	Transgender people	Before and after cross-sectional surveys	Community-led structural intervention	Organisational & technical capacity building of community-based organisations<br>Formation of transgender-run peer support groups	Community-based prevention and linkage to care interventions<br>Outreach through peers, educational materials, social media & facility-based one-on-one counselling<br>Legal, psychosocial & crisis support<br>Trangender people recruited as staff and involved in all capacity building, research & communication activities
Chevrier et al. 'No one was there to care for us': Ashodaya Samithi's community-led care and support for people living with HIV in Mysore, India	2016	India	HIV/AIDS	Commercial sex workers	Qualitative study	Community-led structural intervention	Support group meetings for sex workers living with HIV/AIDS	Volunteers help people access health services & intervene in case of ill treatment or discrimination.<br>Five-bed care home for members released from hospital.<br>Financial support for transport, meals during hospital visits, and funeral rites.<br>Advocacy on behalf of sex workers
Siu et al. Men's Involvement in a Parenting Programme to Reduce Child Maltreatment and Gender-Based Violence: Formative Evaluation in Uganda	2017	Uganda	Child maltreatment & gender-based violence	Women and children	Qualitative study	Participatory group education	21 session community-based parenting programme with a core of 10 single-sex group sessions and 11 mixed-sex group sessions led in a semi-participatory way by a trained facilitator.	None
Hargreaves et al. Process evaluation of the Intervention with Microfinance for AIDS and Gender Equity (IMAGE) in rural South Africa.	2009	South Africa	HIV/AIDS & intimate partner violence	Women	Mixed methods study	Participatory group education & economic strengthening	*IMAGE trial:*<br>Formation & facilitation of combined microfinance & gender training groups	Poverty-focused microfinance<br>Election of 'natural leaders' from within groups to attend external training and develop action plans responding to local priority issues

Author(s) and title	Year	Country focus	Health focus	Target population	Type of article	Type of intervention	Role of community groups	Complementary intervention components
Hatcher et al. Promoting critical consciousness and social mobilization in HIV/AIDS programmes: lessons and curricular tools from a South African intervention	2010	South Africa	HIV/AIDS & intimate partner violence	Women	Qualitative study	Participatory group education & economic strengthening	*IMAGE trial:* Formation & facilitation of combined microfinance & gender training groups	Poverty-focused microfinance Election of 'natural leaders' from within groups to attend external training and develop action plans responding to local priority issues
Pronyk et al. Can social capital be intentionally generated? A randomized trial from rural South Africa	2008	South Africa	HIV/AIDS & intimate partner violence	Women	Mixed methods study	Participatory group education & economic strengthening	*IMAGE trial:* Formation & facilitation of combined microfinance & gender training groups	Poverty-focused microfinance Election of 'natural leaders' from within groups to attend external training and develop action plans responding to local priority issues
Kim et al. Understanding the Impact of a Microfinance-Based Intervention on Women's Empowerment and the Reduction of Intimate Partner Violence in South Africa	2007	South Africa	HIV/AIDS & intimate partner violence	Women	Mixed methods study	Participatory group education & economic strengthening	*IMAGE trial:* Formation & facilitation of combined microfinance & gender training groups	Poverty-focused microfinance Election of 'natural leaders' from within groups to attend external training and develop action plans responding to local priority issues
Gibbs et al. Jobs, food, taxis and journals: complexities of implementing Stepping Stones and Creating Futures in urban informal settlements in South Africa	2014	South Africa	HIV/AIDS & intimate partner violence	Women	Qualitative study	Participatory group education	*Stepping Stones intervention:* Facilitated group sessions where men & women are encouraged to reflect & critically analyse their livelihoods, gender roles & health risks	Participants are provided a journal for private reflection including questions related to group sessions
Gibbs et al. 'Eh! I felt I was sabotaged!': facilitators' understandings of success in a participatory HIV and IPV prevention intervention in urban South Africa	2015	South Africa	HIV/AIDS & intimate partner violence	Women	Qualitative study	Participatory group education	*Stepping Stones intervention:* Facilitated group sessions where men & women are encouraged to reflect & critically analyse their livelihoods, gender roles & health risks	Participants are provided a journal for private reflection including questions related to group sessions

Author(s) and title	Year	Country focus	Health focus	Target population	Type of article	Type of intervention	Role of community groups	Complementary intervention components
Gibbs et al. Reconstructing masculinity? A qualitative evaluation of the Stepping Stones and Creating Futures interventions in urban informal settlements in South Africa	2015	South Africa	HIV/AIDS & intimate partner violence	Women	Qualitative study	Participatory group education	*Stepping Stones intervention:* Facilitated group sessions where men & women are encouraged to reflect & critically analyse their livelihoods, gender roles & health risks	Participants are provided a journal for private reflection including questions related to group sessions
Bhaird. The Complexity of Community Engagement: Developing Staff–Community Relationships in a Participatory Child Education and Women's Rights Intervention in Kolkata Slums	2013	India	Violence against women and girls	Women	Qualitative study	Participatory group education	Mothers of school children invited to form women's groups through which NGO staff facilitated reflection and action	Shikha NGO runs non-governmental schools, women's rights groups, health clinics & vocational training programmes
Falb et al. Creating opportunities through mentorship, parental involvement, and safe spaces (COMPASS) program: Multi-country study protocol to protect girls from violence in humanitarian settings	2016	DRC & Ethiopia	Violence against women and girls	Girls	Study protocol	Participatory group education	Structured, facilitated group sessions in safe spaces focusing on skill-building, self-confidence, friendships, analysis of gender norms & action planning<br><br>Monthly discussion groups for caregivers on gender norms	Capacity building for health service providers & case managers<br>Targeted engagement with partners & family members of girls
Falb et al. Gender norms, poverty and armed conflict in Côte D'Ivoire: engaging men in women's social and economic empowerment programming	2014	Côte D'Ivoire	Intimate partner violence	Women	Qualitative study	Participatory group education & economic strengthening	Facilitated gender dialogue groups for members of village savings & loan associations and their spouses focusing on reflection on financial well-being & gender norms	Village savings and loan associations
Gupta et al. Gender norms and economic empowerment intervention to reduce intimate partner violence against women in rural Côte d'Ivoire: a randomized controlled pilot study	2013	Côte D'Ivoire	Intimate partner violence	Women	Randomised controlled trial	Participatory group education & economic strengthening	Facilitated gender dialogue groups for members of village savings & loan associations and their spouses focusing on reflection on financial well-being & gender norms	Village savings and loan associations

Author(s) and title	Year	Country focus	Health focus	Target population	Type of article	Type of intervention	Role of community groups	Complementary intervention components
Hossain et al. Working with men to prevent intimate partner violence in a conflict-affected setting: A pilot cluster randomized controlled trial in rural Côte d'Ivoire	2014	Côte D'Ivoire	Intimate partner violence	Women	Randomised controlled trial	Participatory group education	Facilitated men's discussion groups focusing on critically reflecting on gender norms & taking action	None
Christofides et al. A cluster randomised controlled trial to determine the effect of community mobilisation and advocacy on men's use of violence in periurban South Africa: study protocol	2018	South Africa	Intimate partner violence	Women	Study protocol	Participatory group education	Regular facilitated community workshops to challenge inequitable & harmful ideas about manhood based on Freirean principles	Formation & training of Community Action Teams who mobilise community members on a voluntary basis around issues of gender transformation.<br>Advocacy to hold government & other duty bearers to account.
Gibbs et al. An individually randomized controlled trial to determine the effectiveness of the Women for Women International Programme in reducing intimate partner violence and strengthening livelihoods amongst women in Afghanistan: trial design, methods and baseline findings	2018	Afghanistan	Intimate partner violence	Women	Study protocol	Participatory group education	Facilitated group sessions with women focusing on savings, health, rights, and networking + training in numeracy, business & vocational skills<br>Support for women with starting Self-Help Groups<br>Facilitated group sessions with men and opinion leaders around gender norms	Monthly conditional cash transfer of USD 10 per month<br>Referral services
Naved et al. A cluster randomized controlled trial to assess the impact of SAFE on spousal violence against women and girls in slums of Dhaka, Bangladesh	2018	Bangladesh	Intimate partner violence	Women	Randomised controlled trial	Participatory group education & structural support	Facilitated group sessions with women and men	Community mobilization group comprising community stakeholders trained & responsible for creating an enabling environment<br>Volunteers recruited to foster change<br>Provision of health & legal services<br>Training of marriage registrars, police, lawyers, and the judiciary.<br>National-level media advocacy

Author(s) and title	Year	Country focus	Health focus	Target population	Type of article	Type of intervention	Role of community groups	Complementary intervention components
Cari Jo Clark et al. Evaluating a multicomponent social behaviour change communication strategy to reduce intimate partner violence among married couples: study protocol for a cluster randomized trial in Nepal	2017	Nepal	Intimate partner violence	Women	Study protocol	Participatory group education & mass media	Facilitated Radio Listening & Discussion Groups meet to critically reflect on gender norms and plan outreach activities	Advocacy with national stakeholders<br>Behaviour change communication through mass media (radio)<br>Training of community & religious leaders
Babalola et al. Impact of a communication programme on female genital cutting in eastern Nigeria	2006	Nigeria	Female genital cutting	Girls	Quasi-experimental study	Participatory group education & mass media	Facilitated community core groups critically reflected on women's reproductive health & developed action plans to improve women's health situation	Advocacy with traditional leaders, religious leaders, local government officials, school authorities, women's groups & ruling councils<br>Discussing female genital cutting at the annual tribal 'Home and Abroad' event<br>Mass media campaign
Diop & Askew. The Effectiveness of a Community-Based Education Program on Abandoning Female Genital Mutilation/Cutting in Senegal	2009	Senegal	Female genital cutting	Girls	Quasi-experimental study	Participatory group education & large-scale inter-village campaign	Participatory group sessions with women on numeracy, literacy, life skills & critical analysis of cultural & socioeconomic conditions<br><br>Women participating in groups encouraged to 'adopt' a friend/relative to share information with from groups	Intervillage meetings to exchange experiences leading to village-wide discussions promoting the idea of abandoning female genital cutting culminating in a large-scale coordinated public declaration by multiple villages on abandoning female genital cutting
Hoque et al. A community-based cluster randomized controlled trial (cRCT) to evaluate the impact and operational assessment of “safe motherhood and newborn health promotion package”: study protocol	2018	Bangladesh	Maternal and child health	Mothers and children	Study protocol	Group discussion & health systems strengthening	Establishment and capacity building of community groups & community support groups	Capacity building and strengthening of health facilities & referral linkages<br>Local level advocacy & planning<br>Maternal, perinatal & community death audits

Author(s) and title	Year	Country focus	Health focus	Target population	Type of article	Type of intervention	Role of community groups	Complementary intervention components
Gram et al. Do Participatory Learning and Action Women's Groups Alone or Combined with Cash or Food Transfers Expand Women's Agency in Rural Nepal?	2018	Nepal	Maternal and child health	Mothers and children	Randomised controlled trial	Participatory group education & economic strengthening	Women's groups practising participatory action & learning	Unconditional food transfers in one intervention arm, unconditional cash transfers in another intervention arm
Gram et al. The long-term impact of community mobilisation through participatory women's groups on women's agency in the household: A follow-up study to the Makwanpur trial	2018	Nepal	Maternal and child health	Mothers and children	Quasi-experimental study	Participatory group education	Women's groups practising participatory action & learning	None
Alcock et al. Community-based health programmes: Role perceptions and experiences of female peer facilitators in Mumbai's urban slums	2009	India	Maternal and child health	Mothers and children	Qualitative study	Participatory group education	Women's groups practising participatory action & learning	None
More et al. Community Mobilization in Mumbai Slums to Improve Perinatal Care and Outcomes: A Cluster Randomized Controlled Trial	2012	India	Maternal and child health	Mothers and children	Randomised controlled trial	Participatory group education	Women's groups practising participatory action & learning	None
Houweling et al. Reaching the poor with health interventions: Programme-incidence analysis of seven randomised trials of women's groups to reduce newborn mortality in Asia and Africa	2015	Nepal, India, Bangladesh, Malawi	Maternal and child health	Mothers and children	Mixed methods study	Participatory group education	Women's groups practising participatory action & learning	Varies with the individual trial included in the set of interventions
Morrison et al. How did formative research inform the development of a women's group intervention in rural Nepal?	2008	Nepal	Maternal and child health	Mothers and children	Mixed methods study	Participatory group education	Women's groups practising participatory action & learning	None

Author(s) and title	Year	Country focus	Health focus	Target population	Type of article	Type of intervention	Role of community groups	Complementary intervention components
Morrison et al. Women's health groups to improve perinatal care in rural Nepal	2005	Nepal	Maternal and child health	Mothers and children	Mixed methods study	Participatory group education	Women's groups practising participatory action & learning	None
Morrison et al. Understanding how women's groups improve maternal and newborn health in Makwanpur, Nepal: a qualitative study	2010	Nepal	Maternal and child health	Mothers and children	Qualitative study	Participatory group education	Women's groups practising participatory action & learning	None
Morrison et al. Utilization and management of maternal and child health funds in rural Nepal	2010	Nepal	Maternal and child health	Mothers and children	Qualitative study	Participatory group education	Women's groups practising participatory action & learning	None
Nair et al. Participatory women’s groups and counselling through home visits to improve child growth in rural eastern India: protocol for a cluster randomised controlled trial	2012	India	Maternal and child health	Mothers and children	Study protocol	Participatory group education & home visits	Women's groups practising participatory action & learning	Home visits with individual families
Prost et al. Women's groups practising participatory learning and action to improve maternal and newborn health in low-resource settings: a systematic review and meta-analysis	2013	Nepal, India, Bangladesh, Malawi	Maternal and child health	Mothers and children	Meta-analysis	Participatory group education	Women's groups practising participatory action & learning	Varies with the individual trial included in the set of interventions
Colbourn et al. Effects of quality improvement in health facilities and community mobilization through women's groups on maternal, neonatal and perinatal mortality in three districts of Malawi: MaiKhanda, a cluster randomized controlled effectiveness trial	2013	Malawi	Maternal and child health	Mothers and children	Randomised controlled trial	Participatory group education & quality improvement at health facilities	Women's groups practising participatory action & learning	One intervention arm also received a health facility quality improvement intervention focusing on conducting small tests of change using Plan-Do-Study-Act cycles, implementing change packages, conducting death reviews & received additional clinical training

Author(s) and title	Year	Country focus	Health focus	Target population	Type of article	Type of intervention	Role of community groups	Complementary intervention components
Azad et al. Effect of scaling up women's groups on birth outcomes in three rural districts in Bangladesh: a cluster-randomised controlled trial	2012	Bangladesh	Maternal and child health	Mothers and children	Randomised controlled trial	Participatory group education	Women's groups practising participatory action & learning	Clinical training for traditional birth attendants<br>Health systems strengthening
Rath et al. Explaining the impact of a women's group led community mobilisation intervention on maternal and newborn health outcomes: the Ekjut trial process evaluation	2010	India	Maternal and child health	Mothers and children	Qualitative study	Participatory group education	Women's groups practising participatory action & learning	Health committees allowing community members to express their opinions about local health services
Rosato et al. Strategies developed and implemented by women's groups to improve mother and infant health and reduce mortality in rural Malawi	2012	Malawi	Maternal and child health	Mothers and children	Qualitative study	Participatory group education & home visits	Women's groups practising participatory action & learning	Training of health workers<br>One intervention arm also received volunteer peer counsellors who identified pregnant women & made home visits
Rosato et al. Women's groups' perceptions of maternal health issues in rural Malawi	2006	Malawi	Maternal and child health	Mothers and children	Qualitative study	Participatory group education & home visits	Women's groups practising participatory action & learning	Training of health workers<br>One intervention arm also received volunteer peer counsellors who identified pregnant women & made home visits
Saville et al. Protocol of the Low Birth Weight South Asia Trial (LBWSAT), a cluster-randomised controlled trial testing impact on birth weight and infant nutrition of Participatory Learning and Action through womens groups, with and without unconditional transfers of fortified food or cash during pregnancy in Nepal	2016	Nepal	Maternal and child health	Mothers and children	Study protocol	Participatory group education & economic strengthening	Women's groups practising participatory action & learning	Unconditional food transfers in one intervention arm, unconditional cash transfers in another intervention arm

Author(s) and title	Year	Country focus	Health focus	Target population	Type of article	Type of intervention	Role of community groups	Complementary intervention components
Wilner et al. Effective delivery of social and behavior change communication through a Care Group model in a supplementary feeding program	2017	Malawi	Maternal and child health	Mothers and children	Qualitative study	Care group model	Community-based health educators held community meetings to facilitate behaviour change	Health educators also conducted home visits, reported on new pregnancies, births & deaths, and disseminated health messages at food distribution points for a supplementary feeding programme to treat moderate acute malnutrition
Aradeon and Doctor. Reducing rural maternal mortality and the equity gap in northern Nigeria: the public health evidence for the Community Communication Emergency Referral strategy	2016	Nigeria	Maternal health	Mothers	Mixed methods study	Group discussion & health systems strengthening	Community-based health volunteers held regular discussion groups to facilitate behaviour change	Involvement of religious ledaers in developing and delivering behaviour change messages through sermons, radio and TV
Brazier et al. The value of building health promotion capacities within communities: Evidence from a maternal health intervention in Guinea	2014	Guinea	Maternal health	Mothers	Quasi-experimental study	Group discussion & health systems strengthening	Village safe motherhood committees & health & hygiene committees lead regular community discussions about maternal health	Village safe mothershood committee members recorded info on pregnancies, births & deaths in their villages<br><br>Health & hygiene committees played a role in oversight of health facility management<br><br>Home visits to monitor maternity
Saha et al. Effect of combining a health program with a microfinance-based self-help group on health behaviors and outcomes	2015	India	Maternal and child health	Mothers and children	Quasi-experimental study	Group discussion & economic strengthening	Facilitated group discussion of health issues in self-help group meetings	Self-help groups engage in income generation, savings & credit provision
Saha, Annear and Pathak. The effect of Self-Help Groups on access to maternal health services: evidence from rural India	2013	India	Maternal health	Mothers	Cross-sectional survey	Group discussion & economic strengthening	Group discussion of health issues in self-help group meetings	Self-help groups engage in income generation, savings & credit provision

Author(s) and title	Year	Country focus	Health focus	Target population	Type of article	Type of intervention	Role of community groups	Complementary intervention components
George et al. Can community action improve equity for maternal health and how does it do so? Research findings from Gujarat, India	2018	India	Maternal health	Mothers	Mixed methods study	Social accountability	Regular community meetings to elicit community concerns, deliver health information, build community ownership & foster collective decision-making<br><br>Monthly meetings convened by volunteers for problem-solving related to community issues	Poster campaign & pictorial banner to support behaviour change communication<br><br>Pictorial check list of maternal health entitlements & services used in community monitoring of health services<br><br>Provision of report cards on health services to women's collectives, ward meetings & medical officers
Gullo et al. Creating spaces for dialogue: a cluster-randomized evaluation of CARE's Community Score Card on health governance outcomes	2018	Malawi	Maternal and child health	Mothers and children	Quasi-experimental study	Social accountability	Regular community meetings to elicit community concerns, deliver health information, build community ownership & foster collective decision-making	Use of Community Score Card in meetings to identify and address obstacles to health coverage, quality and equity<br><br>Separate meetings with men, women, youth, health providers followed by joint meeting with community members, health services and government officials
Mozumdar et al. Increasing knowledge of home based maternal and newborn care using self-help groups: Evidence from rural Uttar Pradesh, India	2018	India	Maternal and child health	Mothers and children	Quasi-experimental study	Group discussion & economic strengthening	Peer health educator gave information to groups at weekly meetings with leaflets for 4 months	Self-help groups engage in income generation, savings & credit provision<br>Local federation of groups

Author(s) and title	Year	Country focus	Health focus	Target population	Type of article	Type of intervention	Role of community groups	Complementary intervention components
Saggurti et al. Effect of health intervention integration within women's self-help groups on collectivization and healthy practices around reproductive, maternal, neonatal and child health in rural India	2013	India	Maternal and child health	Mothers and children	Quasi-experimental study	Group discussion & economic strengthening	Facilitated group discussion of maternal & newborn health issues in self-help group meetings	Self-help groups engage in income generation, savings & credit provision
Akhund and Yousafzai. How successful are women's groups in health promotion and disease prevention? A synthesis of the literature and recommendations for developing countries	2011	Bolivia, Nepal, Pakistan, Guatemala, India, Bangladesh	Maternal and child health	Mothers and children	Narrative review	Participatory group education	Women's groups practising participatory action & learning	Varies with the individual trial included in the set of interventions
Nair et al. Mobilizing women's groups for improved maternal and newborn health: Evidence for impact, and challenges for sustainability and scale up	2012	Nepal, India, Bangladesh	Maternal and child health	Mothers and children	Narrative review	Participatory group education	Women's groups practising participatory action & learning	Varies with the individual trial included in the set of interventions
Rosato et al. Community participation: lessons for maternal, newborn, and child health	2008	Bolivia, India, Ethiopia, Nepal, Bangladesh, Pakistan, Malawi, Tanzania	Maternal and child health	Mothers and children	Narrative review	Primarily participatory group education	Primarily women's groups practising participatory action & learning	Review also included studies on: home-based newborn care, training of mother coordinators to teach other local mothers & community health worker programmes
Marston et al. Community participation for transformative action on women's, children's and adolescents' health	2016	Uganda, Malawi, India, Pakistan, Bangladesh, Nepal	Women, children & adolescent health	Women, children and adolescents	Narrative review	Primarily participatory group education & social accountability	Primarily women's groups practising participatory action & learning and social accountability initiatives	Review also included studies on people-centred health services

**Author(s) and title**	**Year**	**Country focus**	**Health focus**	**Target population**	**Type of article**	**Type of intervention**	**Role of community groups**	**Complementary intervention components**
Farnsworth et al. Community engagement to enhance child survival and early development in low-and middle-income countries: An evidence review	2014	India, Bangladesh, Nepal, Pakistan, Laos, Kenya, South Africa, Zambia, Honduras, Peru, Benin, Tanzania, Vietnam, Ghana, Mexico, Bolivia	Child survival & development	Children	Systematic review	Primarily participatory group education	Primarily women's groups practising participatory action & learning	Review also included studies on interventions relying on other approaches including: diffusion of innovations, health belief mode, most significant change technique, positive deviance, IMCI, socializing evidence participation action & stages of change
Howard-Grabman et al. Factors affecting effective community participation in maternal and newborn health programme planning, implementation and quality of care interventions	2017	Pakistan, India, Bangladesh, Nepal, Peru, Honduras, Indonesia, China, Tanzania, Kenya, Uganda	Maternal and child health	Mothers and children	Review of reviews	Primarily participatory group education	Primarily community groups engaging in reflection & action	Review interventions also included: outreach with community health workers; community-based transportation to health facilities; training of traditional health workers; staskeholder committees; mass media campaigns; community report cards; establishing demonstration households
Muzyamba et al. The role of Community Mobilization in maternal care provision for women in sub-Saharan Africa - A systematic review of studies using an experimental design	2017	Tanzania, Malawi, Kenya, Ethiopia, Angola, Sahel, Nigeria, Sudan, South Africa, Zambia	Maternal and child health	Mothers and children	Systematic review	Primarily participatory group education	Primarily community groups engaging in reflection & action	Review interventions also included: individual peer outreach, outreach by health care workers, involvement of traditional birth attendants, community-based transportation to health facilities
Kumar et al. Pathways from women's group-based programs to nutrition change in South Asia: A conceptual framework and literature review	2017	India, Bangladesh, Nepal, Pakistan	Maternal and child nutrition	Mothers and children	Narrative review	Participatory group education, social accountability, micro-finance & agricultural interventions	Groups engage in participatory learning & action, social accountability, micro-finance or agricultural activities	Review interventions also included a variety of social, economic & behavioural complementary components

Author(s) and title	Year	Country focus	Health focus	Target population	Type of article	Type of intervention	Role of community groups	Complementary intervention components
Cornish et al. The impact of Community Mobilisation on HIV Prevention in Middle and Low Income Countries: A Systematic Review and Critique	2014	India, Tanzania, Uganda, Zimbabwe, South Africa, Thailand, China, Brazil, Ecuador, Dominican Republic	HIV/AIDS	Commercial sex workers, men who have sex with men, adolescents, and general adult populations	Systematic review	Primarily participatory group education & community-led structural interventions	Groups engage in participatory learning & action or form the basis of sex worker collectivization through the formation of community-based organisations	Review interventions also included: training of health workers, condom provision, microfinance, peer outreach, advocacy, STI services, needle exchange
Sebert Kuhlmann, Altman and Galavotti. The importance of community mobilization in interventions to improve sexual, reproductive, and maternal health outcomes: A review of the evidence	2016	India, Pakistan, Bangladesh, Brazil, El Salvador, Guatemala, Burma, Cambodia, Argentina, Tanzania, Zambia, DRC, Australia, Scotland	Sexual, reproductive & maternal health	Women	Narrative review informed by realist review principles	Primarily participatory group education	Primarily women's groups practising participatory action & learning	Review interventions also included: community health worker programmes, mobile clinics, peer education, training of health workers, training of traditional birth attendants, village health committees
Altman, Kuhlmann and Galavotti. Understanding the black box: A systematic review of the measurement of the community mobilization process in evaluations of interventions targeting sexual, reproductive, and maternal health	2015	South Africa, India, Tanzania, Brazil, Dominican Republic, Cambodia, United States	Sexual, reproductive & maternal health	Women	Systematic review	Participatory group education & community-led structural interventions	Groups engage in participatory learning & action or form the basis of sex worker collectivization through the formation of community-based organisations	Review interventions also included: training of health workers, condom provision, microfinance, peer outreach, advocacy, STI services, needle exchange
Berg and Denison. Interventions to reduce the prevalence of female genital mutilation/cutting in African countries	2012	Mali, Egypt, Nigeria, Kenya, Ethiopia, Senegal, Burkina Faso	Female genital cutting	Girls	Systematic review	Primarily participatory group education	Community groups engage in deliberation & action planning	Review interventions also included: identification of role models, classroom education, multimedia campaigns, advocacy, engagement with community leaders

Author(s) and title	Year	Country focus	Health focus	Target population	Type of article	Type of intervention	Role of community groups	Complementary intervention components
Ellsberg et al. Prevention of violence against women and girls: what does the evidence say?	2015	Uganda, Ethiopia, India, Kenya, South Africa, Senegal	Violence against women and girls	Women and girls	Narrative review	Primarily participatory group education	Facilitated groups of women & men engage in reflection & action over gender norms	Review interventions also included: livelihood programmes, support for girls staying in school, self-defence classes, social marketing campaigns, individual volunteer-based activism, advocacy
