# Supplementary material for: Promoting women’s and children’s health through community groups in low-income and middle-income countries: a mixed-methods systematic review of mechanisms, enablers and barriers
Source: BMJ Glob Health. 2019 Dec 5;4(6):e001972. doi: 10.1136/bmjgh-2019-001972 (PMC6936553; doi:10.1136/bmjgh-2019-001972)
Supplement: Supplementary data [file bmjgh-2019-001972supp002.pdf]

Risk of bias assessment – qualitative evidence

Author(s) and title	Year	Did the study aim to explore mechanisms, enablers or barriers?	Was a qualitative methodology appropriate to explore mechanisms, enablers or barriers?	Was the research design appropriate to explore mechanisms, enablers or barriers?	Was the recruitment strategy appropriate to explore mechanisms, enablers or barriers?	Was the data collected in a way that explored mechanisms, enablers or barriers?	Has the relationship between researchers and participants been adequately considered?	Have ethical issues been taken into consideration?	Was the data analysis sufficiently rigorous?	Was there a clear statement of findings?
Biradavolu et al. Structural stigma, sex work and HIV: contradictions and lessons learnt from a community-led structural intervention in southern India	2012	Unclear	Yes	Yes	Unclear	Yes	No	Yes	No	Yes
Kongelf et al. Is Scale-Up of Community Mobilisation among Sex Workers Really Possible in Complex Urban Environments? The Case of Mumbai, India	2015	Yes	Yes	Yes	Yes	Yes	No	Yes	Yes	Yes
Bhattacharjee et al. Understanding the role of peer group membership in reducing HIV-related risk and vulnerability among female sex workers in Karnataka, India	2013	Yes	Yes	Yes	Unclear	Yes	No	Yes	Unclear	Yes
Chevrier et al. 'No one was there to care for us': Ashodaya Samithi's community-led care and support for people living with HIV in Mysore, India	2016	No	Yes	Yes	No	Yes	Yes	Yes	No	Yes

Author(s) and title	Year	Did the study aim to explore mechanisms, enablers or barriers?	Was a qualitative methodology appropriate to explore mechanisms, enablers or barriers?	Was the research design appropriate to explore mechanisms, enablers or barriers?	Was the recruitment strategy appropriate to explore mechanisms, enablers or barriers?	Was the data collected in a way that explored mechanisms, enablers or barriers?	Has the relationship between researchers and participants been adequately considered?	Have ethical issues been taken into consideration?	Was the data analysis sufficiently rigorous?	Was there a clear statement of findings?
Siu et al. Men's Involvement in a Parenting Programme to Reduce Child Maltreatment and Gender-Based Violence: Formative Evaluation in Uganda	2017	Yes	Yes	Yes	Yes	Yes	Yes	Yes	Yes	Yes
Hargreaves et al. Process evaluation of the Intervention with Microfinance for AIDS and Gender Equity (IMAGE) in rural South Africa.	2009	Yes	Yes	Yes	Unclear	Yes	No	Yes	No	No
Hatcher et al. Promoting critical consciousness and social mobilization in HIV/AIDS programmes: lessons and curricular tools from a South African intervention	2010	Yes	Yes	Yes	Unclear	Yes	No	Yes	Yes	Yes
Pronyk et al. Can social capital be intentionally generated? A randomized trial from rural South Africa	2008	Yes	Yes	Yes	Unclear	Yes	No	Unclear	Yes	Yes

Author(s) and title	Year	Did the study aim to explore mechanisms, enablers or barriers?	Was a qualitative methodology appropriate to explore mechanisms, enablers or barriers?	Was the research design appropriate to explore mechanisms, enablers or barriers?	Was the recruitment strategy appropriate to explore mechanisms, enablers or barriers?	Was the data collected in a way that explored mechanisms, enablers or barriers?	Has the relationship between researchers and participants been adequately considered?	Have ethical issues been taken into consideration?	Was the data analysis sufficiently rigorous?	Was there a clear statement of findings?
Kim et al. Understanding the Impact of a Microfinance-Based Intervention on Women's Empowerment and the Reduction of Intimate Partner Violence in South Africa	2007	Unclear	Yes	Yes	Unclear	Yes	Yes	Unclear	No	No
Gibbs et al. Jobs, food, taxis and journals: complexities of implementing Stepping Stones and Creating Futures in urban informal settlements in South Africa	2014	Yes	Yes	Yes	Unclear	Yes	No	Yes	Yes	Yes
Gibbs et al. 'Eh! I felt I was sabotaged!': facilitators' understandings of success in a participatory HIV and IPV prevention intervention in urban South Africa	2015	No	Yes	Yes	Unclear	Yes	Yes	Yes	Yes	Yes

Author(s) and title	Year	Did the study aim to explore mechanisms, enablers or barriers?	Was a qualitative methodology appropriate to explore mechanisms, enablers or barriers?	Was the research design appropriate to explore mechanisms, enablers or barriers?	Was the recruitment strategy appropriate to explore mechanisms, enablers or barriers?	Was the data collected in a way that explored mechanisms, enablers or barriers?	Has the relationship between researchers and participants been adequately considered?	Have ethical issues been taken into consideration?	Was the data analysis sufficiently rigorous?	Was there a clear statement of findings?
Gibbs et al. Reconstructing masculinity? A qualitative evaluation of the Stepping Stones and Creating Futures interventions in urban informal settlements in South Africa	2015	No	Yes	Yes	Unclear	Yes	No	Yes	Yes	Yes
Bhaird. The Complexity of Community Engagement: Developing Staff–Community Relationships in a Participatory Child Education and Women's Rights Intervention in Kolkata Slums	2013	Unclear	Yes	Yes	Yes	Yes	Yes	Unclear	Yes	Yes
Falb et al. Gender norms, poverty and armed conflict in Côte D’Ivoire: engaging men in women’s social and economic empowerment programming	2014	Yes	Yes	Yes	Yes	Yes	Yes	Yes	Yes	Yes

**Author(s) and title**	**Year**	**Did the study aim to explore mechanisms, enablers or barriers?**	**Was a qualitative methodology appropriate to explore mechanisms, enablers or barriers?**	**Was the research design appropriate to explore mechanisms, enablers or barriers?**	**Was the recruitment strategy appropriate to explore mechanisms, enablers or barriers?**	**Was the data collected in a way that explored mechanisms, enablers or barriers?**	**Has the relationship between researchers and participants been adequately considered?**	**Have ethical issues been taken into consideration?**	**Was the data analysis sufficiently rigorous?**	**Was there a clear statement of findings?**
Alcock et al. Community-based health programmes: Role perceptions and experiences of female peer facilitators in Mumbai's urban slums	2009	No	Yes	Yes	Yes	Yes	Yes	Yes	Yes	Yes
Houweling et al. Reaching the poor with health interventions: Programme-incidence analysis of seven randomised trials of women's groups to reduce newborn mortality in Asia and Africa	2015	Yes	Yes	Yes	Yes	Yes	No	Yes	Yes	No
Morrison et al. How did formative research inform the development of a women's group intervention in rural Nepal?	2008	No	Yes	Yes	Unclear	Yes	No	Yes	Yes	Yes
Morrison et al. Women's health groups to improve perinatal care in rural Nepal	2005	Yes	Yes	Yes	Unclear	Yes	No	Yes	Yes	Yes

**Author(s) and title**	**Year**	**Did the study aim to explore mechanisms, enablers or barriers?**	**Was a qualitative methodology appropriate to explore mechanisms, enablers or barriers?**	**Was the research design appropriate to explore mechanisms, enablers or barriers?**	**Was the recruitment strategy appropriate to explore mechanisms, enablers or barriers?**	**Was the data collected in a way that explored mechanisms, enablers or barriers?**	**Has the relationship between researchers and participants been adequately considered?**	**Have ethical issues been taken into consideration?**	**Was the data analysis sufficiently rigorous?**	**Was there a clear statement of findings?**
Morrison et al. Understanding how women's groups improve maternal and newborn health in Makwanpur, Nepal: a qualitative study	2010	Yes	Yes	Yes	Yes	Yes	Yes	Yes	Yes	Yes
Morrison et al. Utilization and management of maternal and child health funds in rural Nepal	2010	Yes	Yes	Yes	Unclear	Yes	Yes	Yes	Yes	Yes
Rath et al. Explaining the impact of a women's group led community mobilisation intervention on maternal and newborn health outcomes: the Ekjut trial process evaluation	2010	Yes	Yes	Yes	Unclear	Yes	No	Yes	Unclear	Yes
Rosato et al. Strategies developed and implemented by women's groups to improve mother and infant health and reduce mortality in rural Malawi	2012	Yes	Yes	Yes	Yes	Yes	No	Yes	Yes	Yes
Rosato et al. Women's groups' perceptions of maternal health issues in rural Malawi	2006	Yes	Yes	Yes	Yes	Yes	No	Yes	Yes	Yes

Author(s) and title	Year	Did the study aim to explore mechanisms, enablers or barriers?	Was a qualitative methodology appropriate to explore mechanisms, enablers or barriers?	Was the research design appropriate to explore mechanisms, enablers or barriers?	Was the recruitment strategy appropriate to explore mechanisms, enablers or barriers?	Was the data collected in a way that explored mechanisms, enablers or barriers?	Has the relationship between researchers and participants been adequately considered?	Have ethical issues been taken into consideration?	Was the data analysis sufficiently rigorous?	Was there a clear statement of findings?
Wilner et al. Effective delivery of social and behavior change communication through a Care Group model in a supplementary feeding program	2017	No	Yes	Yes	Unclear	No	Yes	Yes	Yes	No
George et al. Can community action improve equity for maternal health and how does it do so? Research findings from Gujarat, India	2018	Yes	Yes	Yes	Unclear	Yes	No	Yes	Yes	No
More et al. Community Mobilization in Mumbai Slums to Improve Perinatal Care and Outcomes: A Cluster Randomized Controlled Trial	2012	Yes	Yes	Yes	Unclear	Yes	No	Yes	Unclear	Yes
Saha et al. Effect of combining a health program with a microfinance-based self-help group on health behaviors and outcomes	2015	No	Yes	Yes	Unclear	Yes	No	Yes	Unclear	Yes

Author(s) and title	Year	Did the study aim to explore mechanisms, enablers or barriers?	Was a qualitative methodology appropriate to explore mechanisms, enablers or barriers?	Was the research design appropriate to explore mechanisms, enablers or barriers?	Was the recruitment strategy appropriate to explore mechanisms, enablers or barriers?	Was the data collected in a way that explored mechanisms, enablers or barriers?	Has the relationship between researchers and participants been adequately considered?	Have ethical issues been taken into consideration?	Was the data analysis sufficiently rigorous?	Was there a clear statement of findings?
Aradeon and Doctor. Reducing rural maternal mortality and the equity gap in northern Nigeria: the public health evidence for the Community Communication Emergency Referral strategy	2016	Yes	Yes	Unclear	Unclear	Unclear	No	Unclear	Unclear	Yes
