# Supplementary material for: Promoting women’s and children’s health through community groups in low-income and middle-income countries: a mixed-methods systematic review of mechanisms, enablers and barriers
Source: BMJ Glob Health. 2019 Dec 5;4(6):e001972. doi: 10.1136/bmjgh-2019-001972 (PMC6936553; doi:10.1136/bmjgh-2019-001972)
Supplement: Supplementary data [file bmjgh-2019-001972supp003.pdf]

Risk of bias assessment – quantitative evidence

Author(s) and title	Year	Did the study aim to explore mechanisms, enablers or barriers?	Were the characteristics of the sample adequately described?	Did the study provide estimates of random variability?	Have actual p-values, not cutoffs, been reported?	Were the main findings of the study clearly described?	Was the sample representative of the target population?	Were statistical tests appropriately used?	Were the main measures used valid and reliable?	Was there adequate adjustment for confounding?	Was missing data appropriately taken into account?	Did the study present a mediation or interaction analysis of intervention effect?
Azad et al. Effect of scaling up women's groups on birth outcomes in three rural districts in Bangladesh: a cluster-randomised controlled trial	2012	No	Yes	No	No	Yes	Unclear	No	Unclear	Yes	Unclear	No
Babalola et al. Impact of a communication programme on female genital cutting in eastern Nigeria	2006	No	Yes	Yes	No	Yes	Yes	Yes	Unclear	Partially	No	No
Bhattacharjee et al. Understanding the role of peer group membership in reducing HIV-related risk and vulnerability among female sex workers in Karnataka, India	2013	Yes	Yes	Yes	Yes	Yes	Yes	Yes	Unclear	Unclear	No	No
Blankenship et al. Power, community mobilization, and condom use practices among female sex workers in Andhra Pradesh, India	2008	Yes	Yes	Yes	No	Yes	No	Yes	Unclear	No	No	No

Author(s) and title	Year	Did the study aim to explore mechanisms, enablers or barriers?	Were the characteristics of the sample adequately described?	Did the study provide estimates of random variability?	Have actual p-values, not cutoffs, been reported?	Were the main findings of the study clearly described?	Was the sample representative of the target population?	Were statistical tests appropriately used?	Were the main measures used valid and reliable?	Was there adequate adjustment for confounding?	Was missing data appropriately taken into account?	Did the study present a mediation or interaction analysis of intervention effect?
Brazier et al. The value of building health promotion capacities within communities: Evidence from a maternal health intervention in Guinea	2014	Yes	Yes	Yes	No	Yes	Yes	Yes	Unclear	Partially	No	No
Diop & Askew. The Effectiveness of a Community-Based Education Program on Abandoning Female Genital Mutilation/Cutting in Senegal	2009	No	Yes	No	No	Yes	No	Yes	Unclear	No	No	No
Gaikwad et al. How effective is community mobilisation in HIV prevention among highly diverse sex workers in urban settings? The Aastha intervention experience in Mumbai and Thane districts, India	2012	Yes	No	Yes	No	Yes	Yes	Yes	Unclear	Partially	No	No
George et al. Can community action improve equity for maternal health and how does it do so? Research findings from Gujarat, India	2018	Yes	No	Yes	Yes	No	Unclear	Yes	Yes	Partially	No	No

Author(s) and title	Year	Did the study aim to explore mechanisms, enablers or barriers?	Were the characteristics of the sample adequately described?	Did the study provide estimates of random variability?	Have actual p-values, not cutoffs, been reported?	Were the main findings of the study clearly described?	Was the sample representative of the target population?	Were statistical tests appropriately used?	Were the main measures used valid and reliable?	Was there adequate adjustment for confounding?	Was missing data appropriately taken into account?	Did the study present a mediation or interaction analysis of intervention effect?
Gram et al. Do Participatory Learning and Action Women's Groups Alone or Combined with Cash or Food Transfers Expand Women's Agency in Rural Nepal?	2018	Yes	Yes	Yes	No	Yes	Yes	Yes	Yes	Yes	Yes	No
Gram et al. The long-term impact of community mobilisation through participatory women's groups on women's agency in the household: A follow-up study to the Makwanpur trial	2018	Yes	Yes	Yes	No	Yes	Yes	Yes	Yes	Yes	Yes	No
Guha et al. Risk reduction and perceived collective efficacy and community support among female sex workers in Tamil Nadu and Maharashtra, India: the importance of context	2012	Yes	No	Yes	Yes	Yes	Yes	Yes	Unclear	Partially	No	No

**Author(s) and title**	**Year**	**Did the study aim to explore mechanisms, enablers or barriers?**	**Were the characteristics of the sample adequately described?**	**Did the study provide estimates of random variability?**	**Have actual p-values, not cutoffs, been reported?**	**Were the main findings of the study clearly described?**	**Was the sample representative of the target population?**	**Were statistical tests appropriately used?**	**Were the main measures used valid and reliable?**	**Was there adequate adjustment for confounding?**	**Was missing data appropriately taken into account?**	**Did the study present a mediation or interaction analysis of intervention effect?**
Gupta et al. Gender norms and economic empowerment intervention to reduce intimate partner violence against women in rural Côte d'Ivoire: a randomized controlled pilot study	2013	No	Yes	Yes	Yes	Yes	Yes	Yes	Yes	Yes	Yes	No
Hargreaves et al. Process evaluation of the Intervention with Microfinance for AIDS and Gender Equity (IMAGE) in rural South Africa.	2009	Yes	Yes	No	No	Yes	Unclear	Yes	Yes	No	No	No
Hossain et al. Working with men to prevent intimate partner violence in a conflict-affected setting: A pilot cluster randomized controlled trial in rural Côte d'Ivoire	2014	No	Yes	Yes	No	Yes	Unclear	No	Yes	Yes	No	No
Houweling et al. Reaching the poor with health interventions: Programme-incidence analysis of seven randomised trials of women's groups to reduce newborn mortality in Asia and Africa	2015	Yes	Yes	Yes	Yes	Yes	Yes	Yes	Yes	Partially	No	No

Author(s) and title	Year	Did the study aim to explore mechanisms, enablers or barriers?	Were the characteristics of the sample adequately described?	Did the study provide estimates of random variability?	Have actual p-values, not cutoffs, been reported?	Were the main findings of the study clearly described?	Was the sample representative of the target population?	Were statistical tests appropriately used?	Were the main measures used valid and reliable?	Was there adequate adjustment for confounding?	Was missing data appropriately taken into account?	Did the study present a mediation or interaction analysis of intervention effect?
Kim et al. Understanding the Impact of a Microfinance-Based Intervention on Women's Empowerment and the Reduction of Intimate Partner Violence in South Africa	2007	Yes	Yes	Yes	No	Yes	No	No	Unclear	Yes	No	No
Kuhlmann et al. Investing in communities: Evaluating the added value of community mobilization on HIV prevention outcomes among FSWs in India	2014	Yes	Yes	Yes	Yes	Yes	Yes	Yes	Yes	Partially	No	Yes
Morrison et al. Women's health groups to improve perinatal care in rural Nepal	2005	Yes	No	No	No	Yes	Yes	No	Yes	Yes	Yes	No
Mozumdar et al. Increasing knowledge of home based maternal and newborn care using self-help groups: Evidence from rural Uttar Pradesh, India	2018	No	No	No	Yes	Yes	Unclear	Yes	Yes	Partially	No	No

Author(s) and title	Year	Did the study aim to explore mechanisms, enablers or barriers?	Were the characteristics of the sample adequately described?	Did the study provide estimates of random variability?	Have actual p-values, not cutoffs, been reported?	Were the main findings of the study clearly described?	Was the sample representative of the target population?	Were statistical tests appropriately used?	Were the main measures used valid and reliable?	Was there adequate adjustment for confounding?	Was missing data appropriately taken into account?	Did the study present a mediation or interaction analysis of intervention effect?
Nagarajan et al. Female sex worker's participation in the community mobilization process: Two distinct forms of participations and associated contextual factors	2014	Yes	Yes	Yes	No	No	Yes	Yes	No	No	No	No
Narayanan et al. Monitoring community mobilisation and organisational capacity among high-risk groups in a large-scale HIV prevention programme in India: selected findings using a Community Ownership and Preparedness Index	2012	Yes	Yes	No	No	No	Unclear	No	Unclear	No	No	No
Naved et al. A cluster randomized controlled trial to assess the impact of SAFE on spousal violence against women and girls in slums of Dhaka, Bangladesh	2018	No	Yes	Yes	No	Yes	Yes	Yes	Yes	Yes	No	No

Author(s) and title	Year	Did the study aim to explore mechanisms, enablers or barriers?	Were the characteristics of the sample adequately described?	Did the study provide estimates of random variability?	Have actual p-values, not cutoffs, been reported?	Were the main findings of the study clearly described?	Was the sample representative of the target population?	Were statistical tests appropriately used?	Were the main measures used valid and reliable?	Was there adequate adjustment for confounding?	Was missing data appropriately taken into account?	Did the study present a mediation or interaction analysis of intervention effect?
Pronyk et al. Can social capital be intentionally generated? A randomized trial from rural South Africa	2008	Yes	Yes	Yes	Yes	Yes	No	No	Yes	Yes	No	No
Prost et al. Women's groups practising participatory learning and action to improve maternal and newborn health in low-resource settings: a systematic review and meta-analysis	2013	No	Yes	Yes	Yes	Yes	Yes	Yes	Yes	No	Yes	Yes
Saggurti et al. Community collectivization and its association with consistent condom use and STI treatment-seeking behaviors among female sex workers and high-risk men who have sex with men/transgenders in Andhra Pradesh, India	2013	Yes	Yes	Yes	No	Yes	Yes	Yes	Yes	No	No	No

Author(s) and title	Year	Did the study aim to explore mechanisms, enablers or barriers?	Were the characteristics of the sample adequately described?	Did the study provide estimates of random variability?	Have actual p-values, not cutoffs, been reported?	Were the main findings of the study clearly described?	Was the sample representative of the target population?	Were statistical tests appropriately used?	Were the main measures used valid and reliable?	Was there adequate adjustment for confounding?	Was missing data appropriately taken into account?	Did the study present a mediation or interaction analysis of intervention effect?
Saggurti et al. Effect of health intervention integration within women's self-help groups on collectivization and healthy practices around reproductive, maternal, neonatal and child health in rural India	2013	No	Yes	Yes	Yes	Yes	Yes	Yes	Unclear	Partially	Yes	No
Saha et al. Effect of combining a health program with a microfinance-based self-help group on health behaviors and outcomes	2015	No	Yes	Yes	No	Yes	Unclear	Yes	Yes	Partially	No	No
Saha, Annear and Pathak. The effect of Self-Help Groups on access to maternal health services: evidence from rural India	2013	No	Yes	Yes	No	Yes	Yes	Yes	Yes	Partially	No	No
Shaikh et al. Empowering communities and strengthening systems to improve transgender health: outcomes from the Pehchan programme in India	2016	No	Yes	No	No	Yes	Yes	No	No	No	No	No

Author(s) and title	Year	Did the study aim to explore mechanisms, enablers or barriers?	Were the characteristics of the sample adequately described?	Did the study provide estimates of random variability?	Have actual p-values, not cutoffs, been reported?	Were the main findings of the study clearly described?	Was the sample representative of the target population?	Were statistical tests appropriately used?	Were the main measures used valid and reliable?	Was there adequate adjustment for confounding?	Was missing data appropriately taken into account?	Did the study present a mediation or interaction analysis of intervention effect?
Vejella et al. Community Collectivization and Consistent Condom Use Among Female Sex Workers in Southern India: Evidence from Two Rounds of Behavioral Tracking Surveys	2016	Yes	Yes	Yes	Yes	Yes	Yes	Yes	Yes	No	No	No
Wilner et al. Effective delivery of social and behavior change communication through a Care Group model in a supplementary feeding program	2017	No	No	No	No	No	Unclear	No	No	No	No	No
Beattie et al. Community Mobilization and Empowerment of Female Sex Workers in Karnataka State, South India: Associations With HIV and Sexually Transmitted Infection Risk	2014	Yes	Yes	Yes	Yes	Yes	Yes	Yes	Yes	No	No	Yes
Chakravarthy et al. Community mobilisation programme for female sex workers in coastal Andhra Pradesh, India: processes and their effects	2012	Yes	No	No	Yes	Yes	Unclear	Yes	Yes	No	No	No

Author(s) and title	Year	Did the study aim to explore mechanisms, enablers or barriers?	Were the characteristics of the sample adequately described?	Did the study provide estimates of random variability?	Have actual p-values, not cutoffs, been reported?	Were the main findings of the study clearly described?	Was the sample representative of the target population?	Were statistical tests appropriately used?	Were the main measures used valid and reliable?	Was there adequate adjustment for confounding?	Was missing data appropriately taken into account?	Did the study present a mediation or interaction analysis of intervention effect?
Parimi et al. Mobilising community collectivisation among female sex workers to promote STI service utilisation from the government healthcare system in Andhra Pradesh, India	2012	Yes	Yes	Yes	No	Yes	Yes	Yes	Yes	No	No	No
Aradeon and Doctor. Reducing rural maternal mortality and the equity gap in northern Nigeria: the public health evidence for the Community Communication Emergency Referral strategy	2016	Yes	No	No	No	Yes	Unclear	No	Yes	No	No	No
Gullo et al. Creating spaces for dialogue: a cluster-randomized evaluation of CARE's Community Score Card on health governance outcomes	2018	Yes	Yes	Yes	Yes	Yes	Yes	No	Yes	Partial	Yes	No
