# Supplementary material for: Promoting women’s and children’s health through community groups in low-income and middle-income countries: a mixed-methods systematic review of mechanisms, enablers and barriers
Source: BMJ Glob Health. 2019 Dec 5;4(6):e001972. doi: 10.1136/bmjgh-2019-001972 (PMC6936553; doi:10.1136/bmjgh-2019-001972)
Supplement: Supplementary data [file bmjgh-2019-001972supp004.pdf]

Evidence extraction

Author(s) and title	Year	Evidence concerning mechanisms	Evidence concerning enablers and barriers
Gibbs et al. Reconstructing masculinity? A qualitative evaluation of the Stepping Stones and Creating Futures interventions in urban informal settlements in South Africa	2015	Case stories demonstrated intervention:<br>- provided a safe social space for men to talk about gender norms and learn to express emotions<br>- often improved relationships, particularly communication, but this was not always sustainable<br>- participants often had to choose between losing peer support or reverting to past behaviour<br>- sustainable relationship improvements often only possible after a degree of financial security	<u>*Barrier:*</u> High levels of unemployment meant participants had to spend time continually searched for jobs without getting any.
Gibbs et al. Jobs, food, taxis and journals: complexities of implementing Stepping Stones and Creating Futures in urban informal settlements in South Africa	2014	*Impact of poor attendance:* When participants 'dropped in and out' of the intervention, it became complicated for facilitators to sustain a safe space predicated on trust, as each session would have a new mix of participants with their social dynamics to deal with.	<u>*Ambiguous:*</u> Provision of journals facilitated learning and reflection, but it was often hard to keep them private (e.g. nowhere to keep journal in lodgings or journal might be lost)<br><u>*Barriers:*</u> *Training misread as job opportunity:* Although many arrived without expectations of employment, some became disappointed when they found out there were no job opportunities<br>*Economic barriers:* Inconsistent attendance due to need to seek work - poorly paid and casual - potentially in another city, in order to ensure own financial survival. Although some were able to borrow R20 for the taxi fare to the meeting centre, others struggled.<br>*Gender roles:* Women often borrowed money for the taxi fare from their partner who sometimes effectively 'banned' them from the intervention by refusing to lend them the money
Gibbs et al. 'Eh! I felt I was sabotaged!': facilitators' understandings of success in a participatory HIV and IPV prevention intervention in urban South Africa	2015	*Facilitator interpretation of own role:* Facilitators felt they needed to continually provide input to participants, lead sessions and talking, and position themselves essentially as group leaders. When they had little to say in a session, they felt they had not 'worked hard' enough.<br><br>*Group discussion:* Facilitators recognised the importance of promoting dialogue and discussion and sought to achieve it throughout sessions. Participants actively engaged in group discussion.<br><br>*Encouraging attendance:* Facilitated tried to encourage greater attendance and puncuality by getting participants to agree to start times for sessions, using regular reminder phone calls, and delaying the start of sessions when participants did not arrive, but the strategies did not work.<br><br>*Impact of poor attendance:* Project team felt poor attendance was a major challenge to the intervention which potentially undermined intervention outcomes and offered material incentives to facilitators for achieving high levels of attendance.	<u>*Barriers:*</u> *Facilitator 'expert' mentality:* Facilitators became pre-occupied with ensuring high levels of attendance, prompt arrival of participants, 'correct' answers in group sessions and 'achievement' of behaviour change - all interpreted as markers of success. When they failed to achieve this, they grew frustrated, blamed participants for being lazy, and demanded compliance instead of opening up discussion of the social roots of participants' problems.

Author(s) and title	Year	Evidence concerning mechanisms	Evidence concerning enablers and barriers
Bhaird. The Complexity of Community Engagement: Developing Staff–Community Relationships in a Participatory Child Education and Women's Rights Intervention in Kolkata Slums	2013	*Building relationships with community members:* Gaining trust was a long and unpredictable process, partly built by repeated small-scale interactions, partly fuelled by staff spontaneously responding to emergencies, thus proving their good intent by going beyond official roles.<br><br>*Avoiding community resistance:* Negotiating flexibly and peacefully with communities rather than demanding change, thus conveying respect for local views, using 'well-intentioned deception and manipulation' e.g. branding domestic violence classes as 'literacy classes'.<br><br>*Raising critical consciousness:* Story-telling, problem-tree analysis, gender budgeting and visits to other self-help groups stimulated critical thinking about own situation.<br><br>*Taking action:* Once, women's group became 'too dynamic', vandalised liquor shops and threatened violent men -> backlash of criticism and violence within the community. Otherwise action against domestic violence, lobbying government for a water supply, closing illicit liquor shops and campaigning to obtain electricity.<br><br>*Building social capital:* Group members felt solidarity, unity and new social identity was their biggest achievement.	*Enablers:* Engaging with community members without an air of superiority; consistently delivering the same message in one-on-one and group-level interactions.<br><br>*Barriers:* *Community characteristics:* More difficult to collectivise isolated communities, where women experienced low social cohesion due to female seclusion norms, village exogamy, and competitiveness from poverty and desperation.<br><br>*Poor intervention acceptability:* Negative reaction by men to female 'outsiders' promoting new lifestyles and challenging their dominance from stares and insults to death threats. Suspicion that social change agenda is promoted by a political party looking for votes. Some men forbade wives from attending classes.<br><br>*Staff-community divide:* Lack of confidence among local women to speak to 'well-educated outsiders'. Locals could not relate to language of rights -> protests that classes were a waste of time. Community members quiet and withdrawn, treating staff as guests and outsiders -> difficult to establish open and honest communication. Some staff lacked ethos of open communication and participation and advocated for punitive measures to encourage behaviour change.<br><br>*Divisions between women in the community:* Non-participating women felt neglected and jealous, mothers-in-law felt threatened by daughters-in-law becoming educated, some older women felt insulted to be invited to meetings that they disapproved of.

Author(s) and title	Year	Evidence concerning mechanisms	Evidence concerning enablers and barriers
Siu et al. Men’s Involvement in a Parenting Programme to Reduce Child Maltreatment and Gender-Based Violence: Formative Evaluation in Uganda	2017	*Valuing reflection on gender roles:* Men began to question masculinity norms, share information about parenting with fellow men & learn from them. Confessions & testimonies during sessions suggested parents absorbed key messages & valued learning new communication skills.<br>*Limited changes in relationships:* Men reported experienced changes in relationships including reduced spousal conflict and increased mutual respect. However, some men were not ready to practice conventional maternal behaviours in real life, as opposed to in role plays, or stop restricting their spouses' financial & domestic power. Indeed, husbands tended to police wives' behaviour to accord with programme messages following participation in the programme<br>*Discussing sensitive issues:* Mixed sex sessions occasionally resulted in couples openly discussing sensitive issue in the group, but often helped clarify conflicting perspectives, negative-gendered norms & parenting practices, promoted transparency between the couple and legitimised further discussion of sensitive issues at home.<br>*Learning parenting information:* Men became aware of their limited understanding of child upbringing, but valued learning about positive parenting, bonding & attachment, and alternatives to corporal discipline, although a few found it difficult do without negative tools.	Enablers:<br>*Occupation:* More difficult to recruit & retain men in peri-urban areas, where men worked in a variety of occupations with different time schedules. In rural areas, men worked as fishermen who fished in the evening & returned early in the morning where they had time for the intervention.<br>*Carefully managing composition of sessions:* Fathers valued starting in single-sex sessions before discussing parenting issues with mothers perceived to be more argumentative and assertive; men were also more willling to questions masculinity norms in single-sex than mixed-sex sessions. In the mixed-sex sessions, fathers- and mothers-in-law were not allowed to participate in the same group to minimise reluctance to discuss sensitive issues. Men/women expressed their views to their partner as the 'collective view' of the group, which was less threatening.<br>*Attitudes towards fatherhood:* Men had pre-existing concern with acquiring parenting skills to ensure children should grow up well behaved & welcomed an intervention that recognised them as equal partners in raising their children or even framed them as family heads.<br><br>Barriers:<br>*Entrenched masculinity norms:* Fathers experienced social pressure to conform to conventional masculinity from relatives & wider village membership, which undermined efforts to become a new father.
Kim et al. Understanding the Impact of a Microfinance-Based Intervention on Women's Empowerment and the Reduction of Intimate Partner Violence in South Africa	2007	Qualitative evidence showed:<br>**A.** Initial resistance to discussing domestic violence, particularly older women challenging younger women and expressing views condoning domestic violence, but this dissipated over time<br><br>**B.** Respondents indicated increases in: self-confidence, financial confidence, ability to challenge gender norms, autonomy in decision-making, perceived contribution to the household, quality of partner relationship, social group membership & participation in collective action<br><br>Quantitative evidence showed:<br>Non-significant (p>0.05) increases in all the domains indicated under **B**<br>Non-significant (p>0.05) increases in consumption, membership of a savings group, progressive attitudes to intimate partner violence and lack of controlling behaviour in partner<br>Significant (p<0.05) increases in: household asset value and lack of past year IPV	None provided

Author(s) and title	Year	Evidence concerning mechanisms	Evidence concerning enablers and barriers
Pronyk et al. Can social capital be intentionally generated? A randomized trial from rural South Africa	2008	Quantitative evidence showed:<br>Increased participation in social groups (1.85 aRR, 95% CI 0.95-3.61)<br>Increased belief in community support towards common goals (1.11 aRR, 95% CI 0.38–3.24)<br>Greater perception of community solidarity in times of crisis (1.65 aRR, 95% CI 0.81–3.37)<br>Increased participation in collective action (2.06 aRR, 95% CI 0.92–4.49)<br><br>Qualitative evidence showed:<br>*Trust and solidarity* - group members shared financial and business advice, provided mutual social support (financial, emotional or practical) in times of crisis, broadened their social horizons<br>*Shared norms* evolved through repeated interaction in groups<br>*Vocal leaders* developed group identity & provided inspiration and social influence<br>*Informal action* as women shared insights with children and partners, targeted community groups for awareness-raising, mediated in local conflicts<br>*Collective action* as women organized village workshops, meetings with leadership structures, civic marches, partnerships with local institutions, village committees	Qualitative evidence showed:<br>Loan repayment problems, lack of attendance at meetings, leadership problems - particularly corruption or financial mismanagement, and malicious gossip all undermined solidarity<br><br>Centres with loan repayment problems, leadership challenges or lack of trust between members saw much more limited community mobilization activity<br><br>Women joining groups were generally already familiar with each other as members of other community groups.
Hatcher et al. Promoting critical consciousness and social mobilization in HIV/AIDS programmes: lessons and curricular tools from a South African intervention	2010	*Critical consciousness:* Facilitators created a space where women critically reflected on everyday reality, shared common problems & success stories, felt supported by others with the same problems and developed their own solutions to their problems.<br><br>*Individual action:* Critical consciousness was seen by staff as a catalyst for women to share ideas with the broader community. Participants spoke with household partners and children about formerly taboo subjects like sexuality and also shared information with friends, relatives, church, stokvel & neighbours. Participants also played a role as mediators in family disputes. Participants felt compelled to share information with others and felt it increased their sense of self-efficacy.<br><br>*Collective action:* After learning about social mobilization in a 5-day 'natural leader' training, women conducted meetings with community leaders, held workshops, held public marches, created rape prevention committees & partnered with police and local organizations. This placed many women in leadership roles in their community for the first time.	*Enablers:*<br>*Training and mentoring:* 4-week intensive training and ongoing support & mentorship by managers helped force facilitators to approach group participants as co-learners rather than experts; 5-day 'natural leader' training for group members gave participants a sense of confidence & power.<br><br>*Participatory tools:* Asking participants to analyse roots of ill-health in their communities, probing/asking questions about 'unchallengeable' cultural traditions; conducting role-plays about potentially difficult personal issues; 'grounding' examples in women's daily lives; and emphasising 'going beyond information giving' in group meetings all helped promote critical consciousness<br><br>*Barriers:*<br>*Challenges to the intervention model:* Some facilitators & participants continued to see the function of groups as sharing knowledge with women who were 'blank'; some participants felt frustrated having to implement collective action in their own time using their own financial resources, particularly as they were also expected to run their own micro-finance businesses; local leadership sometimes discouraged activities that did not fall in line with their views, e.g. a march on a clinic feared by the local chief to cause clinic staff to quit.

Author(s) and title	Year	Evidence concerning mechanisms	Evidence concerning enablers and barriers
Hargreaves et al. Process evaluation of the Intervention with Microfinance for AIDS and Gender Equity (IMAGE) in rural South Africa.	2009	Quantitative evidence showed:<br>Clients attended median 8/10 gender training sessions (IQR 5-10). Drop-out from groups 11.1% in first 18 months and 31.2 at 2 years, main reason being trouble with repayments (37%) and death/illness in the household (16%), but not gender training (only 3%). 10% of group members became selected to take part in 'natural leader' training.<br><br>*Client survey showed >80% clients agreed that:* clients learned new things, training had a major impact on their life, they spoke of what they learned to friends & family, others in their loan supported them when there were problems & they participated in organized activities by their local loan centre.<br><br>Qualitative evidence showed:<br>Clients valued focus on communication, new information, social support & increasing confidence from their participation in the programme. Clients felt natural leader training was an additional source of increasing confidence. Acts of individual information sharing were widespread, but not as much collective action.	Quantitative evidence showed:<br>*Predictors of attendance:* Older, married, poorer, less educated women with many children (5+) were more likely to join groups and less likely to drop out after 2 years. Only associations with age were significant at p<0.05.<br><br>Qualitative evidence showed:<br>*Enablers:* Client satisfaction with the programme was enhanced by the use of local trainers.<br>*Barriers:* Sickness, constraints from husbands, childcare and pressures of running a business prevented some women from participating in natural leader training programme. Natural leaders were sometimes insufficiently proactive in planning collective action without the support of programme staff.<br><br>Group members could sometimes not participate in collective action due to: other other family & community responsibilities, lack of monetary incentives for collective action, need to prioritize running a business, social pressure for privacy and 'respectability' & participants' low status in the community due to their extreme poverty.<br><br>Integration between the management structures of the microfinance & gender training components created confusion about roles & responsibilities and negatively affected staff morale
Naved et al. A cluster randomized controlled trial to assess the impact of SAFE on spousal violence against women and girls in slums of Dhaka, Bangladesh	2018	Descriptive quantitative data showed:<br>*Discussion groups:* Staff formed 600 groups (200 per site) - 198 unmarried female, 252 married female, 75 unmarried male & 75 married male groups. Average group size was 15. Proportion eligible community members part of a group was 51% for women and 15% for men. 13 two-hour interactive sessions over a 20-month period conducted separately with female sand males consisting of games, breakout sessions for discussion, role plays & presentation of short plays. On average, women attended 5.8 sessions and men 5.7 sessions.<br>*Community campaigns:* 20-member community mobilisation group comprising representatives from each village selected and supported volunteers to conduct campaigns. In total, 11 rallies, nine video shows, 11 folk music concerts, 11 mobile van campaigns, 3 quiz competition, 6 reflective dialogue sessions, and 8 banner campaigns were organized.	Descriptive quantitative data showed:<br>*Barriers to participation:* 28% of married women & 19% of married men dropped out, mostly due to out-migration (50% for women & 62% for men) and time constraint (35% for both), but also family restrictions (8% married women) and eviction (1%).
Gupta et al. Gender norms and economic empowerment intervention to reduce intimate partner violence against women in rural Côte d'Ivoire: a randomized controlled pilot study	2013	Intention-to-treat analysis showed:<br>Reduced acceptance of wife beating (p<0.01), but no evidence difference in beliefs that a wife had the right to refuse sex with her husband (p>0.05).<br><br>Per-protocol analysis showed:<br>Evidence for reductions in acceptance of wife beating among women with high compliance (p=0.01), but not women with low compliance (p=0.69). No evidence for a difference concerning beliefs about a wife's right to refuse sex with her husband.	None provided

Author(s) and title	Year	Evidence concerning mechanisms	Evidence concerning enablers and barriers
Falb et al. Gender norms, poverty and armed conflict in Côte D'Ivoire: engaging men in women's social and economic empowerment programming	2014	*Improved financial decision-making:* Joint decision-making with wife instead of making decisions on their own leading to reduced wasteful spending, particularly on alcohol.<br><br>*Improved relationships within couples:* Fewer arguments in the household, non-violent ways of resolving conflict & increased respect for wives.<br><br>*Improved social support:* Other men in discussion groups provided social support, particularly advice and guidance regarding household matters, sometimes to non-members as well. One participant felt social cohesion was limited in the group.<br><br>*Challenging inequitable gender norms:* Once program was completed men actively spread information to other men in the community who did not participate.	<u>*Enablers:*</u><br>*Financial benefits of programme:* Generally favourable perceptions of savings and loan programmes for women, particularly among men who also participated in gender dialogue groups, as they were pleased with the additional income; overall support for the programme as a whole from men.<br>*Incentives to participate:* Men were eager to learn financial skills through participation in the gender dialogue groups, as financial prosperity & respect were inextricably linked. However, men also expressed desire to learn to improve spousal relations, better raise children.<br><br><u>*Barriers:*</u><br>*Male resistance:* A small number of men thought groups had no direct benefits for men, increased women's mobility, held up women until late in the night, or felt jealous only women were allowed to be members of the village savings and loan groups
Wilner et al. Effective delivery of social and behavior change communication through a Care Group model in a supplementary feeding program	2017	<u>Quantitative evidence showed:</u><br>Nearly 100% Health care workers reported providing information about optimal child feeding practices to Care Group volunteers; >90% Care Group volunteers reported receiving such information; >90% of caregivers reported receiving such information from Care Group Volunteers and from health care workers.<br><br><u>Qualitative evidence showed:</u><br>Care Group volunteers reported providing a wide range of types of practical and informational social support to pregnant women & children from making home visits to referring women & children to the hospital to advising caregivers on proper sanitation and hygiene.	None provided
Mozumdar et al. Increasing knowledge of home based maternal and newborn care using self-help groups: Evidence from rural Uttar Pradesh, India	2018	<u>Difference-in-difference analysis with propensity score matching showed:</u><br>Self-help group members in the experimental arm reported increases in knowledge on a range of care behaviours in maternal and neonatal health as well as maternal and neonatal danger signs ($p<0.05$)	More educated women showed greater difference in knowledge
Brazier et al. The value of building health promotion capacities within communities: Evidence from a maternal health intervention in Guinea	2014	<u>Multivariable logistic regression showed:</u><br>High Community Support Exposure associated with greater ANC use ($p<0.01$), but not greater institutional delivery or care-seeking for pregnancy complications ($p>0.05$)<br>High ANC Counselling associated with greater institutional delivery ($p<0.05$) & care-seeking for pregnancy complications ($p<0.01$), but not greater ANC use ($p>0.05$)<br>High Community Capacity associated with greater ANC use ($p<0.01$), institutional delivery ($p<0.001$) and care-seeking ($p<0.5$)	None provided

Author(s) and title	Year	Evidence concerning mechanisms	Evidence concerning enablers and barriers
George et al. Can community action improve equity for maternal health and how does it do so? Research findings from Gujarat, India	2018	Quantitative evidence:<br>Substantial increases in awareness of health entitlements (4-48% at baseline depending on type of entitlement -> 64-88% at endline depending on type of entitlement).<br><br>Qualitative evidence:<br>Medical officers and health administrators felt project activities had increased awareness of maternal health, but interviews with community women found some still struggled to articulate rights or accountability as concepts.	Qualitative evidence:<br>*Enablers: NGO mandate:* Staff from NGOs with a mandate to empower collectives of poor women and build capacity for rights-based approaches showed more confidence, required less capacity building & were better able to sustain a cadre of volunteers with low turn-over.<br>*Complementary intervention design:* Participatory development of community report card was essential for local ownership.<br>*Barriers: NGO mandate:* One NGO still saw itself primarily as a service delivery organization & felt discomfort confronting a government with which it had a relationship -> withdrawal from project.
Saha, Annear and Pathak. The effect of Self-Help Groups on access to maternal health services: evidence from rural India	2013	Multivariable logistic regression showed:<br>Presence of a self-help group in a village & having seen or heard health messages were both associated with greater institutional delivery, feeding the child colostrum, knowledge of family planning & use of family planning at $p<0.05$ after adjusting for: education, wealth, presence of a health/sanitation committee, having an accessible community health centre/rural hospital, and being a beneficiary of the maternity cash transfer scheme.	None provided
Saha et al. Effect of combining a health program with a microfinance-based self-help group on health behaviors and outcomes	2015	Difference-in-difference analysis showed:<br>Membership of a self-help group was associated with greater institutional delivery and feeding colostrum to newborns at $p<0.05$. Self-help group members were also more likely to have a toilet at home and less likely to have children with diarrhea, but this was not significant at $p<0.05$.<br><br>Qualitative evidence showed:<br>Group members believed their groups were based on principles of equality, trust, discipline, respect and helping each other. Self-help group meetings acted as a platform for discussing issues that commonly concerned the communities, such as education of children, access to safe drinking water, sanitation, and illness.	Qualitative evidence showed:<br>*Enablers:*<br>*Organizational trust:* Group members showed trust and confidence in the participating organizations due to their association with well-known names (a famous temple trust SKDRDP and trade union SEWA). Organizations also provided material assistance to help solve local water, sanitation and agriculture issues.<br>*Dialogue with stakeholders:* Initial hurdles mitigated through routine feedback to village health workers, dialogue with stakeholders, and training.<br><br>*Barriers:*<br>*Issue size:* Larger issues with road and drainage infrastructure, employment and farming that could not be addressed at the self-help group level, these had to be raised with the village panchayat by group leaders and organizational representatives.<br>*Traditional beliefs:* Tendency to rely on traditional beliefs about health & illness, relying on traditional healers led to delayed care seeking from formal health services and money 'wasted' on seeking care from 'unqualified practitioners'.<br>*Implementation of complementary interventions:* Village health worker dissatisfaction with compensation & frustration with difficulties motivating behaviour change, procedural delays in processing health insurance claims, partner conflict over control over resources from micro-credit

Author(s) and title	Year	Evidence concerning mechanisms	Evidence concerning enablers and barriers
Alcock et al. Community-based health programmes: Role perceptions and experiences of female peer facilitators in Mumbai's urban slums	2009	*Routine activites of group facilitators:* Forming women's groups, persuading women to join groups, manage expectations of material incentives, holding meetings, sharing knowledge<br>*Informal activities of facilitators:* Visiting residents' homes, maintaining relationships, offering advice, seeking out health info to relay to the community, helping women access services<br><br>*Facilitator interpretation of own role:* Group facilitators felt they had to become 'a friend' to women in the community to establish trust to facilitate behaviour change; facilitators felt behaviour change was most effectively promoted by combining examples from own experiences, 'new' knowledge from training, and picture cards to illustrate their point.	<ins>*Enablers:*</ins><br>*Credibility/trust by women in the community was enhanced by:*<br>Group facilitators being older, having children, being friends with women in the community, being perceived as knowledgable and experienced, and having a professional status (enhanced by the use of picture cards).
Rath et al. Explaining the impact of a women's group led community mobilisation intervention on maternal and newborn health outcomes: the Ekjut trial process evaluation	2010	*Stories and games:* Group facilitators made cause-effect linkages explicit using stories and games in meetings. Group members disseminated stories about pregnancy and delivery illustrating both health problems and strategies for solving them during community meetings. Facilitators and members narrated an estimated 976 new stories during 3-year study period.<br><br>*Inclusiveness of groups:* Important decision-makers besides pregnant women themselves - such as men, relatives and frontline government workers - sometimes attended group meetings and supported women.<br><br>*Health advocacy:* Group members supported local village health committees, involved community health workers in discussions about entitlements to health services, made home visits, arranged transport for emergencies, provided financial help from a group emergency fund, and counselled relatives of pregnant women.	<ins>*Enablers:*</ins><br>*Intervention acceptability enhanced by:*<br>Recruitment and training of local facilitators, use of locally appropriate discussion materials, flexibility in timing and content of meetings<br>*Trust in group facilitators was enhanced by:* Being from the study area, respecting local practices, and knowing local languages.<br>*Learning and planning within groups was enhanced by:* The use of locally appropriate discussion materials (picture cards, stories, and participatory games), structured phase-wise content of the meeting cycle, emphasis on collective problem-solving<br><br><ins>*Barriers:*</ins><br>Initial difficulties building rapport with tribal communities, managing expectations of financial gain, contending with dominant group members, managing cancellations during festivals and cultivation periods, managing presence of men during sensitive discussions and rare disruptions from non-group members, maintaining participation during internal village conflicts, handling possible opposition by in-laws and TBAs who felt meetings went against traditional practices, health services were sometimes difficult to access due to remoteness of villages, poor access to transport and bad road conditions
Morrison et al. How did formative research inform the development of a women's group intervention in rural Nepal?	2008	None provided	<ins>*Barriers:*</ins><br>*Intervention design:* Government-run female community health volunteers were tasked with running monthly women's groups, but these were often not functioning, as volunteers found it difficult to sustain a regular group without regular training, supervision, an agenda and health education tools.<br>*Sociocultural context:* Low decision-making power for women, gender inequality, cultural beliefs that pregnancy, childbirth and the period afterwards are shameful and polluting; poor utilization of traditional birth attendants by community members and reluctance of TBAs to be involved in births

Author(s) and title	Year	Evidence concerning mechanisms	Evidence concerning enablers and barriers
Morrison et al. Women's health groups to improve perinatal care in rural Nepal	2005	*Deliberation:* Picture card game was used to guide groups towards matching problems with strategies<br>Groups nominated members to present findings or perform socio-dramas during community planning meetings; issues of health care underutilization & poor service delivery were often raised.<br><br>*Information dissemination:* Groups carried picture cards home to non-attendees to educate them; a supervisor initiated discussion about newborn health in a bus; a group organized a quiz with a community<br><br>*Strategies favoured:* 69 groups started mother & child health funds; 23 groups made clean home delivery kits; 42 groups started stretcher schemes; some groups held large video screenings using a MIRA film.<br><br>*Improved linkages with the health system:* In many cases group members brokered links between the community & the health system, e.g. in 12 wards, women's group invited by local health institution to select new community health volunteers & traditional birth attendants.	*Enablers:*<br>Local support from political groups, local health staff & men enabled active groups; prioritised problems reflected local perceptions of seriousness & frequency of problems and were different in each community; discussions were livelier & planning more productive when local health personnel & chairmen attended.<br>*Barriers:*<br>77 out of 111 women's groups implemented strategies & 100 continued to meet to discuss perinatal health, but rest did not meet due to: unstable security situation, lack of support/ hostility from local leaders, husbands or health workers, and general lack of interest.<br>Local community volunteer supposed to run group meetings, but was often left unsupervised & unsupported, which made it difficult for her to run meetings.<br>*Not modifiers:*<br>No specific formula for an active women's groups including ethnic & social homogeneity, geography, distance from a market area, caste composition
Morrison et al. Understanding how women's groups improve maternal and newborn health in Makwanpur, Nepal: a qualitative study	2010	*Learning about health:* Women's groups felt to be a source of support & a place for learning and sharing knowledge; group facilitators also felt they had personally developed.<br>*Gaining confidence:* Reticence or embarrassment/*laj* decreased making women ready to openly discuss; group discussion & strategy implementation helped build confidence; members sometimes challenged household norms, for example stigma against intercaste marriage<br>*Spreading information about good practice:* Group members disseminated information about maternal & neonatal health to other women either informally or through community meetings; health workers reported increased knowledge about cleanliness at birth; groups disseminated information while distributing resources (such as clean delivery kits) to families<br>*Planned strategies:* Stretcher schemes, revolving funds, clean delivery kits & use of picture card games; these enabled groups to mobilise community resources & strengthen social networks, which strengthened community capacity to address problems	None provided

Author(s) and title	Year	Evidence concerning mechanisms	Evidence concerning enablers and barriers
Morrison et al. Utilization and management of maternal and child health funds in rural Nepal	2010	*Flexibility in borrowing:* Group rules about joining & leaving, borrowing & repayment and contribution levels were often inconsistent/contradictory suggesting a lack of clarity & flexibility in lending; lending often at the discretion of the committee member holding the fund<br><br>*Increasing motivation:* Group members motivated by fund mobilization process, as it demonstrated to them they were knowledgeable, confident & could understanding planning for the future. Local availability & immediacy freed members from dependence on landlords, friends & neighbours. Other stakeholders also felt groups & savings were a source of personal development, learning & autonomy.	*Trust:* Fund management, contribution & borrowing usually restricted to women's group members & close families due to fear & suspicion of mismanaging money/'outsiders' making inappropriate withdrawals; mutual trust & understanding was felt to be important for fund success; lenders could not lend to the poorest, because the risk was too high.<br><br>*Funds discouraging membership:* Although funds encouraged some women to join, stronger evidence that it discouraged group members - all members paid the same amount regardless of socioeconomic status, there were many savings & credit schemes in the study area & funds were often insufficient to meet health needs, as they were limited by the poverty of group members; lack of contribution due to mistrust of fund management, poverty, or scepticism about the impact of the commmunity; lack of contribution from better-off community members due to the perception that the groups were exclusive to poorer people
Houweling et al. Reaching the poor with health interventions: Programme-incidence analysis of seven randomised trials of women's groups to reduce newborn mortality in Asia and Africa	2015	<u>Qualitative data indicated:</u><br>Many groups ran savings/emergency funds and supported arranging transport at the time of delivery.	<u>Quantitative data indicated:</u> Women who were poorer, less educated, older and not primigravid more likely to attend group meetings, but these associations were not consistently significant at p<0.05 across trial sites and intervention years.<br><br><u>Qualitative data indicated:</u><br>*Attendance improved by:* Group facilitators going door-to-door to invite and persuade women to attend; convening meetings where poorer people reside; perception groups were not exclusive to a socioeconomic group; use of simple and fun discussion tools; holding meetings close to women's homes and at convenient times.<br><br>*Attendance hindered by:* Wealthier, educated women feeling groups were beneath them; primigravid women feeling unconfident discussing reproductive health; perception groups not relevant for childless women or women not yet pregnant; older group members directly excluding younger women; belief young women were vulnerable to evil spirits in group meetings; female seclusion norms; fear young women would gossip about her 'new' household, learn bad habits from other community members, or become too independent

Author(s) and title	Year	Evidence concerning mechanisms	Evidence concerning enablers and barriers
Rosato et al. Women's groups' perceptions of maternal health issues in rural Malawi	2006	Quantitative evidence showed:<br>Among 172 groups, 78 different health problems were identified, most commonly anemia (87% of groups), malaria (80%), retained placenta (77%), obstructed labour (76%), malpresentation (71%), antepartum hemorrhage (70%), pre-eclampsia (56%), miscarriage (41%) & eclampsia (35%).<br><br>During prioritisation, anaemia was most commonly ranked among the top five problems (47% of groups), then malpresentation (43%) & retained placenta (43%), then obstructed labour (40%), then postpartum hemorrhage (40%), then malaria (38%), then antepartum hemorrhage (25%), then eclampsia (13%) & pre-eclampsia (8%).<br><br>Qualitative evidence showed:<br>Women appreciated that identification of problems was an essential first step; groups felt they were in the best position to identify problems, because they knew them through own experiences and those of friends & relatives; groups chose own criteria on which to judge importance, most commonly whether it lead to death and whether it had recently been experienced by many women in the community.	Quantitative evidence showed: HIV/AIDS was not among the top ten health problems identified (only 31% of groups) and only 5% prioritised it among their top five priorities.<br><br>Qualitative evidence showed: HIV/AIDS was considered a complex illness that lacked coherence in its symptoms or the problems that it caused; there was a taboo around open discussion of the issue; the disease was considered untreatable and therefore fruitless to address.
Rosato et al. Strategies developed and implemented by women's groups to improve mother and infant health and reduce mortality in rural Malawi	2012	Quantitative evidence showed:<br>11 strategies were initially identified to solve prioritised maternal & neonatal health problems, 21 strategies were implemented in practice, on average groups implemented 8 strategies each (range 0-18) with 1679 strategies over 197 groups during the year 2009.<br><br>Most commonly identified strategies: health education (96% of groups), bicycle ambulances (88%), training of traditional birth attendants (71%), dimba garden cultivation (61%), distribution of insecticide-treated bednets (60%), mobile clinics (48%), small-scale income generating activities (37%). 52% of groups ultimately implemented group-revolving funds.<br><br>Qualitative evidence showed strategies were operationalised in varied and sophisticated ways, often requiring group members to lobby government, health centre or private donors for staff & resources for their strategy as well as establishing committees to oversee such resources.	None provided

Author(s) and title	Year	Evidence concerning mechanisms	Evidence concerning enablers and barriers
More et al. Community Mobilization in Mumbai Slums to Improve Perinatal Care and Outcomes: A Cluster Randomized Controlled Trial	2012	Qualitative evidence showed:<br>Women expressed a need for information on health and health care when the project began, as existing slum women's groups and action groups focused on single issues on an ad hoc basis; group members were also enthusiastic about acquiring new knowledge.<br><br>Group members made substantial efforts to reach out to other local women, particularly by providing information on antenatal & newborn care, referring women to health providers, and occasionally accompanying them.<br><br>Key collective activities undertaken by groups were: attempts to create local awareness, extending support to other women, negotiating with civic authorities for amenities, mobilizing and sharing resources. However, in general, efforts were more individual than collective.<br><br>Quantitative evidence showed:<br>Facilitators formed 244 groups based in individual alleys, a median of ten per cluster. A mean of 5 women attended each meeting (range 2-20) and individual women attended 15 meetings each (range 1-50). A sample of 235 members reported helping 1,372 other distinct women, i.e. on average one woman reached out to six others. However, sharp drop in group membership during later phases that attempted to broker collective strategizing; 150 out of 244 were sustained to the end of the cycle & member numbers dropped from 2,948 to 656.	Qualitative evidence showed:<br>*Barriers:* Members were often reluctant to circulate, and the small size of homes restricted attendance & women also needed to do piecework and care for their families; collective action was challenging, as women's desire for knowledge was tempered by time pressure and immediate concerns, such as insecurity of tenure; membership was informal and withdrawal easy when participants felt their commitment would be too onerous.
Gram et al. Do Participatory Learning and Action Women's Groups Alone or Combined with Cash or Food Transfers Expand Women's Agency in Rural Nepal?	2018	Descriptive quantitative data showed:<br>Substantial increases in group attendance in intervention arms combining women's groups with cash or food transfers (from 34% of pregnant women to >90%)<br><br>Multivariable regression showed:<br>No evidence for impact on household agency in the domains work outside the home, household chores & health seeking at $p<0.05$, but evidence for impact in the domain of group participation ($p<0.01$).<br><br>No evidence for impact on decision-making on large household purchases, food preparation & serving or own pregnancy at $p<0.05$. No evidence for an impact on overall rating of empowerment or perceptions of being an agent in one's own life at $p<0.05$.	None provided
Gram et al. The long-term impact of community mobilisation through participatory women's groups on women's agency in the household: A follow-up study to the Makwanpur trial	2018	Multivariable regression showed:<br>No evidence at $p<0.05$ for a positive impact on household agency in work outside the home, household chores, health seeking and group participation across a range of specifications, but evidence for a negative impact on agency in group participation ($p<0.05$) under some specifications.<br><br>No evidence for impact on decision-making on large household purchases or overall rating of empowerment at $p<0.05$, but evidence for a negative impact on overall empowerment under some specifications ($p<0.05$).	None provided

Author(s) and title	Year	Evidence concerning mechanisms	Evidence concerning enablers and barriers
Azad et al. Effect of scaling up women's groups on birth outcomes in three rural districts in Bangladesh: a cluster-randomised controlled trial	2012	Process evaluation data showed:<br>Main strategies implemented by women's groups: creation of emergency funds managed by the groups, awareness-raising of maternal & newborn health issues during meetings and in the community, fostering effective communication with health-care providers through meetings with group representatives. Strategies changed over time and varied between groups.	None provided
Saggurti et al. Effect of health intervention integration within women's self-help groups on collectivization and healthy practices around reproductive, maternal, neonatal and child health in rural India	2013	Compared to the comparison group, difference-in-difference regression showed:<br>Increase in collective efficacy (adjusted DID estimate 16.9, p<0.01, 95% CI 7.5 to 26.2)<br>Decrease in collective agency (-5.4, p>0.05, -11.6 to 0.6)<br>Decrease in collective action (-0.7, p>0.05, -9.5 to 8.1)<br>Increase in SHG accompaniment for ANC (7.7, p<0.05, 1.0 to 14.4)<br>Increase in SHG visit 2 days after delivery (31.8, p<0.01, 24.1 to 39.3)<br>Increase in receipt of RMNCH information from SHG member outside of meetings (45.4, p<0.01, 37.6 to 53.2)	None provided
Guha et al. Risk reduction and perceived collective efficacy and community support among female sex workers in Tamil Nadu and Maharashtra, India: the importance of context	2012	*Collective efficacy and community support:*<br>Meeting attendance was associated with greater collective efficacy & community support in all areas (p<0.05) except Mumbai (p>0.05); belonging to a self-help group was associated with greater collective efficacy & community support in non-Chennai Tamil Nadu (p<0.05); belonging to a sex worker collective was associated with greater collective efficacy & community support in non-Chennai Tamil Nadu and Mumbai (p<0.05), but not non-Mumbai Maharashtra.<br><br>*Consistent condom use with clients:*<br>Meeting attendance was associated with greater condom use in non-Chennai Tamil Nadu and non-Mumbai Maharashtra (p<0.05), but not Tamil Nadu or Mumbai. Belonging to a self-help group was associated with condom use in non-Chennai Tamil Nadu and Mumbai (p<0.05), but not the other two areas. Belonging to a sex worker collective was associated with reduced condom use in Mumbai (p<0.05), but not in non-Chennai Tamil Nadu or non-Mumbai Mahrashtra.	None provided
Saggurti et al. Community collectivization and its association with consistent condom use and STI treatment-seeking behaviors among female sex workers and high-risk men who have sex with men/transgenders in Andhra Pradesh, India	2013	High *collective efficacy* was associated with improvements (p<0.05) in: consistent condom use with occasional and regular clients, STI treatment, high self-efficacy for condom use & high self-efficacy for utilization of government health services.<br><br>High *collective agency* was only associated with improvements in STI treatment and self-efficacy for utilization of government health services (p<0.05), not other outcomes. High *collective action* was associated with improvements in all outcomes (p<0.05), except STI treatment.<br><br>Adjustment for self-efficacy suggested collective efficacy was independently associated with consistent condom use with clients, but affected STI treatment through self-efficacy	None provided

Author(s) and title	Year	Evidence concerning mechanisms	Evidence concerning enablers and barriers
Vejella et al. Community Collectivization and Consistent Condom Use Among Female Sex Workers in Southern India: Evidence from Two Rounds of Behavioral Tracking Surveys	2016	Between 2010 and 2012, *collective efficacy* and *collective agency* decreased, but *collective action* increased (p<0.001); self-efficacy for condom use with clients & partners and actual consistent condom use with clients and partners all increased (p<0.001).<br><br>Significant interaction (p<0.05) between *collective efficacy* and time of survey, with greater impact on condom use with regular & occasional clients at endline compared to baseline. Significant interaction (p<0.05) between *collective agency* and time of survey, with greater impact on consistent condom use with regular & occasional clients at endline compared to baseline. Significant interaction (p<0.05) between *collective action* and time of survey, with greater impact on consistent condom use with occasional clients, but not concerning regular clients (p>0.05)<br><br>Adjusting for self-efficacy in condom use did not change the interaction coefficients above meaningfully. Significant interactions (p<0.05) were found with time of survey on impact of self-efficacy with greater impact of *collective agency* at endline, but lower impact of *collective action* at endline compared to baseline. No evidence for interaction for *collective efficacy* (p>0.05).	None provided

Author(s) and title	Year	Evidence concerning mechanisms	Evidence concerning enablers and barriers
Gaikwad et al. How effective is community mobilisation in HIV prevention among highly diverse sex workers in urban settings? The Aastha intervention experience in Mumbai and Thane districts, India	2012	Multivariable logistic regression showed:<br>High collective efficacy *to address goals* was associated with greater self-efficacy in condom use with clients & partners, but lower ability to turn away clients refusing to use a condom ($p<0.05$)<br>High collective efficacy *to address a problem* was associated with greater self-efficacy in condom use with clients & patners, and higher ability to turn away clients refusing condoms ($p<0.05$)<br><br>High collective efficacy *to address goals* was associated with greater ability to stand up against police, madams, powerful stakeholders & service providers, and greater confidence in supporting and receiving support from fellow sex workers ($p<0.05$).<br>High collective efficacy *to address a problem* was associated with greater ability to stand up against powerful stakeholders & service providers and greater confidence to support fellow sex workers ($p<0.05$), but not standing up to police/madams or receiving help from others ($p>0.05$)<br><br>High collective efficacy *to address goals* or *to address a problem* were associated with greater confidence in saying no to clients, when unwilling to have sex, disclosing identity as a sex worker to service providers, travelling alone, giving advice to neighbours and other sex workers & voicing an opinion in front of a large group, although association with disclosing identity only significant ($p<0.05$) for collective efficacy to address a problem<br><br>High collective efficacy *to address goals* or *to address a problem* were both associated with graeter overall empowerment ($p<0.05$)	None provided
Kuhlmann et al. Investing in communities: Evaluating the added value of community mobilization on HIV prevention outcomes among FSWs in India	2014	Path analysis showed:<br>% unpaid volunteers on committees improved self-confidence in giving advice, collective efficacy for goals, collective identity & social cohesion ($p<0.05$), but not collective efficacy for problem, collective agency or self-efficacy in condom use ($p>0.05$).<br><br>Only social cohesion related to consistent condom use with clients ($p<0.01$) and only collective efficacy for goals related to perceived fair treatment at service centres ($p<0.01$).	None provided

Author(s) and title	Year	Evidence concerning mechanisms	Evidence concerning enablers and barriers
Shaikh et al. Empowering communities and strengthening systems to improve transgender health: outcomes from the Pehchan programme in India	2016	Before-and-after analysis showed:<br>Access to condoms, lubricants and a wide variety of health, legal and support services increased from baseline to endline, as did coverage of HIV education outreach and referral for HIV testing and counselling (p<0.01), but no evidence for increase in ART counselling (p=0.297).<br><br>More transgender people felt empowered with advocacy skills for services and possessed information about sex reassignment surgery at endline compared to baseline (p<0.001).<br><br>Endline survey showed:<br>Most respondent confident that transgender people and CBOs could work together to address a problem (74%), speak collectively for their rights (83%) & protect each other from harm (79%)<br><br>Many were willing to seek health services in places where health workers were aware of their transgender identity (58%), were confident they would take an HIV test regularly (63%), had participated in a public event identifying them as transgender (58%)<br><br>Many had also been helped by other transgender participants or a CBO when last arrested (61%) or had been helped by transgender peers or a CBO when partner became violent (46%)	None provided
Narayanan et al. Monitoring community mobilisation and organisational capacity among high-risk groups in a large-scale HIV prevention programme in India: selected findings using a Community Ownership and Preparedness Index	2012	Descriptive quantitative data showed:<br>Between 2008-2009 and 2009-2010, median scores among community-based groups *for female sex workers* for leadership (39->37%) & resource mobilisation (29->29%) did not change noticeably; scores for governance (24->29%), programme management (31->39%), decision-making (14->21%), engagement with the state (18->36%) & engagement with wider society (10->14%) increased; scores for networking (24->19%) decreased. At least 22 CBGs were not considered ready to transition to independence based on a self-imposed threshold limit.	*Enablers:*<br>CBG members frequently reported in surveys that they join groups to address issues of rights, social entitlements and stigma.

Author(s) and title	Year	Evidence concerning mechanisms	Evidence concerning enablers and barriers
Nagarajan et al. Female sex worker's participation in the community mobilization process: Two distinct forms of participations and associated contextual factors	2014	Bivariable logistic regression showed:<br>Participation in collective spaces was consistently associated with collective identity, community identity & collective agency, but not collective ownership or perceived vulnerability to the police at p<0.05 across Andhra Pradesh, Tamil Nadu & Maharashtra<br><br>Multivariable logistic regression showed:<br>Participation in collective spaces was consistently predicted by collective identity & collective ownership at p<0.05 across states<br><br>Participation in collective spaces was not consistently predicted at p<0.05 across states by community identity, collective agency, participation in public spaces or perceived vulnerability to the police<br><br>Participation in public spaces was consistently predicted at p<0.05 by collective agency, collective ownership, participation in collective spaces & perceived vulnerability to the police across states.<br><br>Participation in public spaces was not consistently predicted by collective identity & community identity across states at p<0.05.	None provided
Blankenship et al. Power, community mobilization, and condom use practices among female sex workers in Andhra Pradesh, India	2008	Bivariable logistic regression showed:<br>Greater collective identity, collective efficacy & collective agency associated with more intense programme exposure at p<0.001<br><br>Multivariable logistic regression showed:<br>Consistent condom use was predicted by greater collective identity, collective efficacy & collective agency at p<0.01, but association no longer significant (p>0.05) after adjusting for control over type of sex, money charged, living environment and any programme exposure; any programme exposure did predict consistent condom use after adjusting for all the above<br><br>No evidence for interaction between programme exposure and collective identity or efficacy in determining condom use at p<0.05, but evidence for positive interaction with collective agency	None provided

Author(s) and title	Year	Evidence concerning mechanisms	Evidence concerning enablers and barriers
Biradavolu et al. Structural stigma, sex work and HIV: contradictions and lessons learnt from a community-led structural intervention in southern India	2012	*CBO formation as a strategy to challenge stigma:* Community-based organisations were instrumental in challenging sex worker stigma, because sex workers could now identify with an organisation meant specifically for their occupational group.<br><br>*Collective action:* NGO organised public events where CBO members visibly identified themselves as sex workers and engaged in police & media advocacy.<br><br>*Towards self-sustainability:* Training largely non-literate CBO members in organisational management required considerable inputs from NGO staff in order to teach them disciplinary norms, agenda creation, documentation and implementation of decisions, as well as regular elections and rotation of leaders & training in monitoring, performance evaluation & public speaking. Trained Social Change Agents participated in, took decisions on & felt ownership of implementation activities and developed confidence in their abilities to run an organisation. After two years, sex workers felt confident about taking over control of the organisation.	*Barriers:*<br>*Poor synergy with complementary services:* Social Change Agents' bold self-identification as sex workers helped challenge stigma, but undermined their ability to convince peers to seek services at the local health clinic who wanted to remain secret & anonymous. Some Social Change Agents tried to circumvent this by meeting sex workers in secret, outside of public locations. It was suggested that treating all women, not just sex workers, at the clinic would remove this obstacle, but this was felt to be contrary to the organisation's mandate to challenge sex worker stigma.
Bhattacharjee et al. Understanding the role of peer group membership in reducing HIV-related risk and vulnerability among female sex workers in Karnataka, India	2013	Propensity score matching showed:<br>Evidence for effect of group/collective membership on reductions in experience of violence, police extortion, lack of identification document, gonorrhea/chlamydia and syphilis, but not lack of consistent condom use, forced sex or HIV infection at $p<0.05$ after adjusting for socio-demographic confounders<br><br>Qualitative evidence showed:<br>Group members displayed more confidence dealing clients due to their ability to enlist the support of other sex workers and inform other group members of bad clients -> better ability to negotiate condom use, avoid forced sex and violence and negotiate a fair price; however, group members still felt helpless in dealing with police violence<br><br>Group members were less dependent on exploitative credit, better able to move out of debt and better able to refuse unprotected sex where collectives had initiated savings and loan schemes<br><br>Membership of a collective was not a factor influencing knowledge around condoms and STIs, accessibility to condoms/clinical service, perceptions of having a right to practice sex work or willingness to disclose own sex worker identity to others	None provided

Author(s) and title	Year	Evidence concerning mechanisms	Evidence concerning enablers and barriers
Kongelf et al. Is Scale-Up of Community Mobilisation among Sex Workers Really Possible in Complex Urban Environments? The Case of Mumbai, India	2015	None provided	Barriers:<br>*Diversification of sex work:* Continuous targeting of female sex workers created awareness of link between sex work and HIV in the general population, leading to client preferences for hidden sex. As sex work shifted from brothel- to street-, phone- & home-based work, it became less visible, less networked & more autonomous, which made it difficult to reach & mobilise women.<br>*Stigma against sex worker identity:* Programme objective to maximise coverage and verify this through monitoring made it obvious that sex workers were its main target population, leading some to shun the programme who felt the 'sex worker' label was stigmatising<br>*Fraught relations with power brokers:* Madams, pimps & agents sometimes wanted to keep sex workers hidden. Limited power to stop police raids of brothels led some sex workers & pimps to believe that the intervention was informing the police & bringing on raids, which reduced trust.<br>*Residential mobility:* Female sex workers moved around to earn more & avoid police raids, which led to challenges with service provision, follow-up, and achievement of behaviour change.<br>*Demographic heterogeneity:* Women migrated to Mumbai from across state & national boundaries leading to competition, rivalry, mistrust & animosity between sex workers of different backgrounds, which made it challenging to bring community-based groups working with sex workers of different backgrounds together under a single organisation.<br>*Performance monitoring:* Pressure to demonstrate performance shifted focus towards the people who were the easiest to reach and most visible & the outcomes that were measured (condom use), inhibiting ability to respond to realities on the ground, which inhibited programme participation & led to the creation of unsustainable community-based organisation, which existed "on paper" only.
Babalola et al. Impact of a communication programme on female genital cutting in eastern Nigeria	2006	Before-and-after analysis showed:<br>*For Enugu state:* Significant change in all ideational factors in anti-FGC direction among men and women at $p<0.05$, except for self-reported encouragement of others not to cut, which was only significant among men.<br><br>Dose-effect response to exposure to mass media campaign and community mobilisation with greater effect of the two interventions combined than either alone on all ideational factors except personally approving of FGC at $p<0.05$. Impact on the latter was not reported.<br><br>Attenuation in association between programme exposure and intention not to cut after adjusting for ideational factors indicating possible mediating role of ideational factors.<br><br>*For Ebonyi state:* No evidence for change in any ideational indicator at $p<0.05$.	None provided

Author(s) and title	Year	Evidence concerning mechanisms	Evidence concerning enablers and barriers
Diop & Askew. The Effectiveness of a Community-Based Education Program on Abandoning Female Genital Mutilation/Cutting in Senegal	2009	*Increased awareness:* Participating women (87%) and men (36%) identified TOSTAN as main source of info on FGC; 92% women shared information from the programme & 90% observed public discussions about FGC compared to 0% in comparison villages. Awareness of at least two consequences of FGC increased among male (11->80%) and female (7->83%) participants. Nonparticipating women (47%) and men (45%) could also mention at least two consequences at endline. Awareness in comparison groups increased 3->25% for women and 14->21% for men.<br><br>*Changes in attitudes:* Significant reductions in women approving of FGC, having a partner approving of FGC & wanting daughters to be cut among both participant, non-participant & non-intervention women at p<0.001, but greater reductions among women in intervention villages. 85% of participant & 64% of non-participant women who disapproved of FGC at endline stated they had changed their minds due to the TOSTAN programme, but so did 12% of women disapproving of FGC in the control area. Similar reductions in intention to cut daughter among participant, non-participant & non-invention men w/ greater reductions in intervention villages.<br><br>*Changes in outcome:* Significant increase in uncut girls aged 0-10 according to their mothers at p<0.001 among participant and non-participant women, but no evidence for a change in control	<u>Descriptive quantitative data showed:</u><br>*Barriers to participation:* When the program started, 64% women & 50% men who originally expressed an interest in participating actually did. Most common reason for non-participation: for men that there was no financial compensation, for women that classes were overcrowded, they got sick, became pregnant, or their husbands forbade them to join. Only 69% women & 57% men who participated completed all four program modules.
Chevrier et al. 'No one was there to care for us': Ashodaya Samithi's community-led care and support for people living with HIV in Mysore, India	2016	Initial support group meetings rapidly made clear that everyone living with HIV regardless of their involvement with sex work encountered discrimination and stigma. For many, joining the organization was the moment, they realised many others encountered the same problems that they encountered.	None provided
Beattie et al. Community Mobilization and Empowerment of Female Sex Workers in Karnataka State, South India: Associations With HIV and Sexually Transmitted Infection Risk	2014	<u>Individual-level impact on individual and collective power:</u> Sex workers who had high exposure to the intervention – defined as being a member of a peer group or collective or attending a drop-in centre or NGO meeting – were more likely to report feeling a strong sense of unity with other sex workers, attend a rally where they could be identified as a sex worker, stand up to or negotiate with someone on behalf of a sex worker, and feel confident negotiating condom use in adjusted analyses (p<0.05).<br><br><u>Outcome analysis:</u><br>No clear trends between intervention exposure and levels of STIs or HIV at district level. No evidence for association between intervention exposure and HIV, syphilis or HSV-2 infection in biological samples (p>0.05), but evidence for chlamydia/gonorrhoea (p<0.05) as a combined outcome.	None reported
Chakravarthy et al. Community mobilisation programme for female sex workers in coastal Andhra Pradesh, India: processes and their effects	2012	3-5 years later, all community-based organisations (CBOs) had higher average scores on the Community Ownership and Preparedness Index, a measure of CBO capacity, but none of the CBOs were rated as highly sustainable and capable of self-governance.	None reported

Author(s) and title	Year	Evidence concerning mechanisms	Evidence concerning enablers and barriers
Parimi et al. Mobilising community collectivisation among female sex workers to promote STI service utilisation from the government healthcare system in Andhra Pradesh, India	2012	Collective efficacy, collective agency, and collective action were all associated with increased self-efficacy for service utilization from health facilities and increased actual STI treatment from the same (p<0.05).	None reported.
Aradeon and Doctor. Reducing rural maternal mortality and the equity gap in northern Nigeria: the public health evidence for the Community Communication Emergency Referral strategy	2016	<u>Qualitative evidence:</u><br>Intervention communities established a savings scheme for emergency loans or outright gifts, an emergency transport scheme, and a blood donors group to accompany women referred to a facility. Communities also put social pressure on husbands and families of pregnant women to support institutional delivery and skilled birth attendance.<br><br><u>Quantitative evidence:</u><br>Knowledge of maternal and child health related care and care seeing practices increased from 15-26% at baseline to 60-91% at endline two years later. 81-84% of community members were aware of savings, transport and blood donation scheme at endline. Private attitudes and social norms concerning husband's responsibility to support women during pregnancy both improved.	<u>Qualitative evidence:</u><br>Intervention was effective because community volunteers spread knowledge and commitment to action using communication tools specifically designed for low-literacy populations.
Gullo et al. Creating spaces for dialogue: a cluster-randomized evaluation of CARE's Community Score Card on health governance outcomes	2018	<u>Instrumental variable analysis found:</u><br>Participation in the intervention did not increase trust in health workers, mutual responsibility, power sharing or collective efficacy, but furthered awareness of Community Action Groups & Safe Motherhood Committees.<br><br>*Among women who indicated meetings had taken place between health providers and communities in their village*, participation increased scores on trust in health workers, mutual responsibility, participation in negotiated spaces, joint monitoring and transparency, equity and quality, effective collective action & community help (p<0.05), but not collective efficacy or power sharing (p>0.05). In the same population, the above had a mixed association with receipt of home visits from health workers, use of modern family planning methods, and satisfaction with services.	None reported
